# Differentially expressed microRNAs in peripheral blood cell are associated with downregulated expression of IgE in nonallergic childhood asthma

**DOI:** 10.1038/s41598-023-33663-5

**Published:** 2023-04-19

**Authors:** Jyh-Hong Lee, Li-Chieh Wang, Yu-Tsan Lin, Yao-Hsu Yang, Hsin-Hui Yu, Ya-Chiao Hu, Bor-Luen Chiang

**Affiliations:** 1 Department of Pediatrics, National Taiwan University Hospital and National Taiwan University College of Medicine, 8 Chung-Shan South Road, Taipei, 10002 Taiwan, Republic of China; 2 Graduate Institute of Clinical Medicine, National Taiwan University College of Medicine, Taipei, Taiwan, Republic of China

**Keywords:** Immunology, Biomarkers, Pathogenesis

## Abstract

Childhood asthma is a heterogeneous disease characterized by chronic airway inflammation, leading to a broad range of clinical presentations. Nonallergic asthma is asthma without allergic sensitization. Both clinical manifestations and immunopathological mechanisms of nonallergic childhood asthma were rarely investigated. We aimed to compare the clinical features between nonallergic and allergic childhood asthma and apply microRNA to explore the underlying mechanism of nonallergic childhood asthma. We enrolled 405 asthmatic children (76 nonallergic, 52 allergic with total IgE < 150 IU/mL and 277 allergic with total IgE > 150 IU/mL). Clinical characteristics were compared between groups. Comprehensive miRNA sequencing (RNA-seq) was performed using peripheral blood from 11 nonallergic and 11 allergic patients with elevated IgE, respectively. Differentially expressed miRNA (DEmiRNA) were determined with DESeq2. Kyoto Encyclopedia of Genes and Genomes (KEGG) and Gene Ontology (GO) analysis was performed to determine functional pathways involved. Publicly available mRNA expression data was applied to investigate the predicted target mRNA networks via Ingenuity Pathway Analysis (IPA). The average age of nonallergic asthma was significantly younger (5.614 ± 2.743 *vs* 6.676 ± 3.118 years-old). Higher severity and worse control were more common in nonallergic asthma (two-way ANOVA, *P* < 0.0001). Long-term severity was higher, and intermittent attacks persisted in nonallergic patients. We identified 140 top DEmiRNAs based on false discovery rate (FDR) *q*-value < 0.001. Forty predicted target mRNA gene were associated with nonallergic asthma. The enriched pathway based on GO included Wnt signaling pathway. IgE expression was predicted to be downregulated by a network involving simultaneous interaction with *IL-4*, activation of *IL-10* and inhibition of *FCER2*. Nonallergic childhood asthma were distinct in their younger age, higher long-term severity and more persistent course. Differentially expressed miRNA signatures associate with downregulation of total IgE expression and predicted target mRNA genes related molecular networks contribute to canonical pathways of nonallergic childhood asthma. We demonstrated the negative role of miRNAs involved in regulating IgE expression indicating differences between asthma phenotypes. Identification of biomarkers of miRNAs could contribute to understand the molecular mechanism of endotypes in nonallergic childhood asthma, which can potentially allow delivery of precision medicine to pediatric asthma.

## Introduction

Asthma is a heterogeneous disease characterized by chronic airway inflammation and variable airway remodeling, giving rise to a broad range of clinical presentations, treatment responses, and natural histories across the disease course^1^. Phenotypes are therefore characteristics that can be directly observed and measured (either biochemically or physically)^2^. Our recognition of the heterogeneity and complexity of childhood asthma phenotypes has led to many in-depth studies of the underlying pathophysiological and/or immunological mechanisms of childhood asthma. We usually classify asthma by endotypes, which refers to different disease subtypes based on common physiological pathway or pathological mechanisms^3^. Endotype research has led to the discovery of disease-specific biomarkers that can aid in clinical diagnosis, disease classification, phenotyping, patient management and predicting long-term prognosis^4^.

The earliest classification of asthma subtypes was extrinsic and intrinsic subtypes. Clinical asthma is initially divided into allergic (atopic) and nonallergic (nonatopic) asthma^5^. The most often methods we had later used for asthma classification is based on the association with T helper type 2 (Th2) cell-mediated inflammatory allergic sensitization^6^. Recent investigations could identify three types (types 1, 2, and 3) of cell-mediated immune responses based on specific lineages of effector T cells and innate lymphocytes (ILCs). These three types of immune response could explain the heterogeneity of asthma and influence the composition of granulocyte infiltration within the airways, resulting in four inflammatory phenotypes of asthma (eosinophilic, neutrophilic, pauci-granulocytic, and mixed-granulocytic)^7,8^.

The nonallergic form predominates among older people (typically seen in those aged > 30 years) and exhibits a female predominance^9–11^. The generally accepted definition of nonallergic asthma is asthma without allergic sensitization in skin prick or in vitro IgE tests to a panel of local allergens (at a minimum a panel of perennial allergens); the total serum IgE level is typically normal or low^12^. In total, 10–33% of asthmatics have nonallergic asthma or asthma that does not seem to be associated with allergic sensitization^10,12^. Nonallergic asthma is often more severe than allergic asthma and may be less responsive to standard therapy^12^. Early onset allergic asthma is most prevalent in childhood and young adulthood and that atopy and its severity are important features of childhood asthma. There is evidence to support the existence of nonallergic childhood asthma distinct from the allergic subtype^6,13^. Few studies have compared nonallergic and allergic childhood asthma and the clinical manifestations of nonallergic childhood asthma were rarely characterized.

MicroRNAs (miRNAs), one of small noncoding RNAs, are single-stranded RNAs with around 19–24 nucleotides, targeted on 3ʹ untranslated messenger RNA (mRNA) region leading to degradation, translational inhibition, or posttranslational downregulation of gene expression and thus regulate the expression of protein-coding genes^14^. The expression of specific microRNAs differ between asthma patients and healthy controls and the expression of some microRNAs were increased in patients with higher asthma severity^15^. The observations on the specific roles of microRNAs in asthma highlight their regulatory effect on allergic inflammation in the airway, Th1/Th2 polarization, immune reactivity, asthma disease severity, and clinical response to therapy^14,16,17^. The precise mechanisms that regulate nonallergic asthma, traditionally referred to as intrinsic asthma, remain enigmatic^18^. Application of miRNA in exploring the underlying mechanism of nonallergic childhood asthma is at needed.

We first compared the clinical features of nonallergic and allergic childhood asthma, with particular emphasis on baseline characteristics and long-term asthma severity. We here explored miRNA profiling between nonallergic patients and allergic patients to understand the underlying mechanism.

## Material and methods

### Asthma patients diagnosis

Asthma definitions were based on children with clinical features and characteristics suggestive of asthma were diagnosed according to the criteria of the National Asthma Education and Prevention Program Expert Panel Report 3 (EPR-3)^19^ and GINA^20^ and on the combination of the following criteria: (i) *Symptoms criteria*: asthma symptoms (wheezing and/or cough symptoms and respiratory distress) in the last 12 month; (ii) *Medical diagnosis*: asthma diagnosed by a pediatrician/physician; (iii) *Medication criteria*: asthma medication (based on current use of inhaler treatment) in the previous 12 months; (iv) *Functional criteria*: reversibility of an increase in either forced expiratory volume in 1 s (FEV_1_) > 12% or predicted FEV_1_ > 10% after inhalation of a short-acting β-agonist (SABA); or peak expiratory flow (PEF) variability ≥ 20%. Although most childhood asthma starts early in life, the majority of infants and young children who have wheezing episodes do not progress to persistent asthma. Early wheezing phenotypes were excluded according to GINA^20^. We also excluded children with concurrent congenital heart or lung diseases, primary immunodeficiency, infectious disease or genetic diseases.

From September 1 2004 to April 30 2021, we enrolled 405 children from the Department of Pediatrics, National Taiwan University Hospital (NTUH). Total serum IgE, allergen sensitization and peripheral blood miRNA data were collected at the time of recruitment. All patients were treated with Fluticasone propionate (FP) or an equivalent inhaled corticosteroid (ICS) for a minimum of three months, with varying dosages as low (100–250 μg/day), medium (> 250–500 μg/day), or high (> 500–1000 μg/day). Clinical data including (1) frequency of daytime symptoms, (2) frequency of nighttime symptoms, (3) peak expiratory flow (PEF) (predicted %), (4) PEF variability (%), (5) asthma severity, and (6) asthma control during interviews and follow-ups were measured according to EPR-3^19^ and collected as previous described^21^. The study was approved by the Institutional Review Board and Research Ethics Committee of the National Taiwan University Hospital and adhered to the tenets of good clinical practice and principles of the Declaration of Helsinki. Informed consent was obtained from all participants and/or their legal guardians.

Clinical data (age, average asthma severity, average asthma control, total serum IgE, and blood eosinophil percentage) were compared statistically among the groups by using the Mann–Whitney *U*-test (GraphPad Prism 9).

### Serum total and allergen-specific IgE levels

Total serum IgE levels were measured using the CAP FEIA system (Pharmacia, Uppsala, Sweden) according to the manufacturer’s instructions. The data were calibrated using the World Health Organization standard for total serum IgE, within the range 2–5000 IU/mL. Allergen sensitization was determined using the MAST Optigen test (Hitachi Chemical Diagnostics, Mountain View, CA). Demographic and baseline characteristics were obtained as descriptive statistics. IgE values > 5000 IU/mL were assigned values of 5000 IU/mL.

### Nonallergic vs allergic

Children were defined as having allergic asthma (AA) when they had a positive allergen-specific IgE level (positive sensitization; allergen specific IgE > 0.35 IU/mL) and/or increased total serum IgE level in accordance with clinical symptoms (history of cough, recurrent wheezing, recurrent difficulty breathing, and recurrent chest tightness). Allergic asthma patients with total IgE levels > 150 IU/mL were classified as elevated IgE allergic asthma (elevated IgE AA). Allergic asthma patients with total IgE levels < 150 IU/mL were classified as low IgE allergic asthma (low IgE AA). Symptoms tend to recurrent or worsen at night or at early morning, exposure to allergens and air irritants, changes in weather, hard laughing or crying, and stress. Diagnosis also included a family history of eczema, hay fever, asthma or atopic disease.

Nonallergic asthma (NA) is defined when patient has no personal or family history of allergic symptoms and with whom allergic sensitization cannot be identified (by skin prick or in vitro allergen specific-IgE tests with allergen specific IgE < 0.35 IU/mL) to a panel of local allergens, including dust mites, danders, feathers, molds, grasses/trees, foods, cockroach mix, and latex. Serum total IgE levels should be low (< 150 IU/mL)^11–13,22,23^. NA was characterized by symptoms induced primarily by the common cold, viral infection, respiratory tract infections, cold air or changes in climate temperature, smoke and air pollution or exercise but not environmental allergens^13^.

### Longitudinal pattern analysis of asthma severity

The median total IgE level of our 277 elevated IgE AA patients was 535 IU/mL (interquartile range: 333.5–1037 IU/mL). To evaluate the impact of total serum IgE level on the longitudinal trends of childhood asthma severity, we divided the 405 patients into the following groups based on their IgE levels: IgE < 150 IU/mL (including 76 NA + 52 low IgE AA), IgE 150–535 IU/mL (138 elevated IgE AA), and IgE > 535 IU/mL (139 elevated IgE AA). From all three groups, we selected patients with follow-up durations > 5 years. Asthma severity were classified as: intermittent (1 point), mild persistent (2 point), moderate persistent (3 point), and severe persistent (4 point)^19^. The locally weighted scatterplot smoothing (LOWESS) function of Prism software (version 8; GraphPad Software Inc., La Jolla, CA) was used to fit curves without any need for model selection^24^. We use the linear regression method to compare the statistic difference between LOWESS curves.

### Sequencing of miRNAs with RNA-seq

#### Library preparation and sequencing

Peripheral blood samples were randomly obtained from 11 patients with NA and 11 patients with elevated IgE AA adjusted for age and sex (supplement Table S1). Total RNA was extracted from peripheral blood white blood cells using the miRNeasy Extraction Kit (Qiagen) according to the manufacturer’s protocol. RNA quantity was assessed by NanoDrop 2000 spectrophotometers (Thermo Scientific), and RNA mass and integrity was assessed using Agilent 2100 Bioanalyzer system and the Agilent RNA 6000 Nano Kit (Agilent Technologies) at the site of RNA extraction and again at sequencing unit. A total of 22 RNA samples with RNA integrity number (RIN) ≥ 7 proceeded to the library construction for RNA-Seq data generation.

A total of 1.2 μg of total RNA per sample was used as input material for the small RNA library. Sequencing libraries were generated using the TruSeq Small RNA Library Prep Kit (Illumina) following the manufacturer’s recommendations and index codes were added to attribute sequences to the samples. Briefly, 3'- and 5'-adapters were specifically ligated to the 3'- and 5'-ends of small RNAs. Next, first-strand cDNA was synthesized using SuperScript II Reverse Transcriptase. PCR amplification was performed using 2 × PCR Master Mix and the PCR products were resolved in a BluePippin 3% agarose gel. DNA fragments of 120–160 bp were recovered and dissolved in 15 μL of double-distilled water. Library quality was assessed using the Agilent Bioanalyzer 2100 system and DNA High-Sensitivity Chips. The qualified libraries were then sequenced on Illumina NextSeq 500 platform with 75 bp single-end reads. Raw reads were processed using NGS QC toolkit.

#### miRNA-Seq read mapping and quantification of transcript expression (FPKM/RPM)

Unaligned data were written to a FASTQ file, and then clean reads were annotated with miRBase to identify known miRNAs using Bowtie 1 (v1.3.1)^25^. We use tools FastQC (https://www.bioinformatics.babraham.ac.uk/projects/fastqc/) to create a report of sequence quality. From the mapped sequences, the number of reads per annotated genes are counted. For each step, quality reports are aggregated using MultiQC^26^.

MiRNA expression levels are expressed as reads per million (RPM). We used DESeq2 (version 1.26.0) for normalization and differential expression analysis^27^. DESeq2 performs an internal normalization where geometric mean is calculated for each gene across all samples. The read counts for a gene in each sample is then divided by this mean. The median of these ratios in a sample is the size factor for that sample. This procedure corrects for library size and RNA composition bias. Log_2_FoldChange formula is: log2FC = Log_2_(specific miRNA expression of NA)-Log_2_(specific miRNA expression of elevated IgE AA). The DEmiRNAs between the two childhood asthma groups were identified at *P*-levels < 0.05 after Benjamini–Hochberg correction for multiple comparisons. Heatmaps were generated by uploading the DEmiRNA data to ClustVis 2.0 (https://biit.cs.ut.ee/clustvis/). The accession number for the sequencing and expression data of miRNA reported in this paper is the Gene Expression Omnibus (GEO) series accession number, GSE222775.

### Publicly available RNA-seq data

Public available mRNA RNA-seq expression data (Log_2_FC and *P* value) downloaded from the NCBI Short-Read Archive (SRA) database from the publication by Raedler et al. for CD4^+^ T cells as well as peripheral blood mononuclear cells (PBMCs) from NA, AA and healthy controls were downloaded from the Gene Expression Omnibus (BioProject PRJNA175377: series accession GSE40887 and BioProject PRJNA175378: series accession GSE40888, respectively)^13^.

### Ingenuity pathway analysis

miRNA expression profiles were analyzed by Ingenuity Pathway Analysis (IPA) software (Qiagen), using the *target filters*, *core analysis,* and *pathway explore* functions. Analytical condition:

- Data Source: ALL
- Confidence Level: Experimentally observed
- Species: Human
- Tissure & Cell line: Tissues and primary cells
- Mutation: All
- Relation Types: All
- Publication Date Ranges: All
- Node Types: All
- Disease: Immunological disease, inflammatory diseases/responses, Respiratory diseases
- Biofluids: All

Direct interaction pathways, nodes, and predicted target mRNAs were overlaid with the DEmiRNAs of NA and elevated IgE AA to plot network diagrams. The molecular activity prediction (MAP) tool enables us to predict the upstream and/or downstream effects of activation or inhibition of molecules in a network given one or more neighboring molecules with “known” activity. We used the expression levels of the top DEmiRNAs and publicly available mRNA RNA-seq expression data (Log_2_FC and *P* value) as inputs in IPA network analysis to visualize the overall effect on network. Only the overlapped mRNA genes between the predicted target mRNA gene of DEmiRNAs and the mRNA genes from publicly available expression data (GSE40887 and GSE40888, respectively) will be included for constructing the molecular network.

MiRNAs control biological responses by regulating targeted gene expression. Therefore, Kyoto Encyclopedia of Genes and Genomes (KEGG)^28^ and Gene Ontology (GO) analysis of predicted targeted mRNAs can reveal the role of of DEmiRNAs. Enrichment analysis of predicted target mRNAs was additionally performed to determine the major biological functions and pathways based on KEGG and GO database.

### Ethics approval and consent to participate

The study was approved by the Institutional Review Board and Research Ethics Committee of the National Taiwan University Hospital and adhered to the tenets of good clinical practice and principles of the Declaration of Helsinki. Informed consent was obtained from all participants and/or their legal guardians.

## Results

### Patient characteristics

Seventy-six patients with NA, 52 patients with low IgE AA, and 277 patients with elevated IgE AA were identified^22^. Their demographic, asthma severity, asthma control, total serum IgE level, sensitization status, and comorbidity (allergic rhinitis [AR] and atopic dermatitis [AD]) data are compared in Table 1. We showed the average age at enrolment of both children with NA (5.614 ± 2.743 years-old) and those with low IgE AA (5.177 ± 2.552 years-old) was significantly younger than that of those with elevated IgE AA (6.676 ± 3.118 years-old) (Table 1; supplemental Fig. S1). We explored the association between age at enrollment and average asthma severity in patient with NA and found that the association were not significant (Pearson *r* =—0.1994, *P* value = 0.084). We also demonstrated that the total serum IgE levels of both children with NA (60.72 ± 92.38 IU/mL) and those with low IgE AA (89.35 ± 38.68 IU/mL) was significantly lower than that of those with elevated IgE AA (909.4 ± 972.7 IU/mL) (Table 1; supplemental Fig. S1). We diagnosed 76 (18.8%) children with NA, in agreement with the fact that NA is observed in 10–33% of asthmatics (Table 1).

**Table 1:** Descriptive characteristics of 405 patients with childhood asthma.

	Patients with NA (n = 76)	Patients with low IgE AA (n = 52)	Patients with elevated IgE AA (n = 277)
Age at enrollment: years, mean ± SD (range)	^¶^5.614 ± 2.743** (2.00–14.13)	§5.177 ± 2.552*** (1.42–12.0)	6.676 ± 3.118 (1.42–17.0)
F:M (ratio)	37:39	20:32	113:164
Average asthma severity (mean ± SD )^†^	^¶^2.051 ± 0.670*	1.921 ± 0.440	1.878 ± 0.478
Average asthma control (mean ± SD)^‡^	^¶^1.681 ± 0.385^#^	1.693 ± 0.434	1.564 ± 0.3349
Total serum IgE (IU/mL; mean ± SD) (range)	^¶^60.72 ± 92.38**** (2.00–127)	§89.35 ± 38.68**** (6.72–149)	909.4 ± 972.7 (152–5000)
Peripheral blood eosinophil (%; mean ± SD) (range)	^¶^2.838 ± 2.341**** (0.10–11.2)	§3.887 ± 2.353** (0.10–9.9)	5.936 ± 4.569 (0.10–26.0)
Sensitization rate (%)			
Dust mites	0	87.27	95.47
Danders	0	16.36	23.77
Molds	0	3.64	5.66
Grass	0	1.82	5.28
Foods	0	30.91	50.19
Cockroach	0	12.73	30.94
Latex	0	1.82	0.85
Comorbidity (%)			
Allergic rhinitis (AR)	0	56.36	50.19
Atopic dermatitis (AD)	0	1.82	3.02
AR + AD	0	20	34.72
Nil (asthma only)	100	21.82	12.07
Inhaled corticosteroids (ICS) dose (μg/day)^Ψ^	221.7 ± 86.10****	197.9 ± 70.65^#^	198.7 ± 93.65

*NA* nonallergic asthma, *low IgE AA* allergic asthma with total IgE levels < 150 IU/mL, *elevated IgE AA* allergic asthma with total IgE levels > 150 IU/mL.

^†^Asthma severity were classified as: intermittent (1 point), mild persistent (2 point), moderate persistent (3 point), and severe persistent (4 point).

^‡^Asthma control were classified as: well controlled (1 point), not well controlled (2 point) and very poorly controlled (3 point).

^¶^Nonallergic *vs* IgE > 150 IU/mL.

^§^IgE < 150 IU/mL *vs* IgE > 150 IU/mL.

^#^Borderline (*P* < 0.1); *: *P* < 0.05; **: *P* < 0.01; ***: *P* < 0.001; ****: *P* < 0.0001.

^Ψ^Fluticasone propionate equivalent according to GINA 2019, Box 3–6.

**Figure 1:**
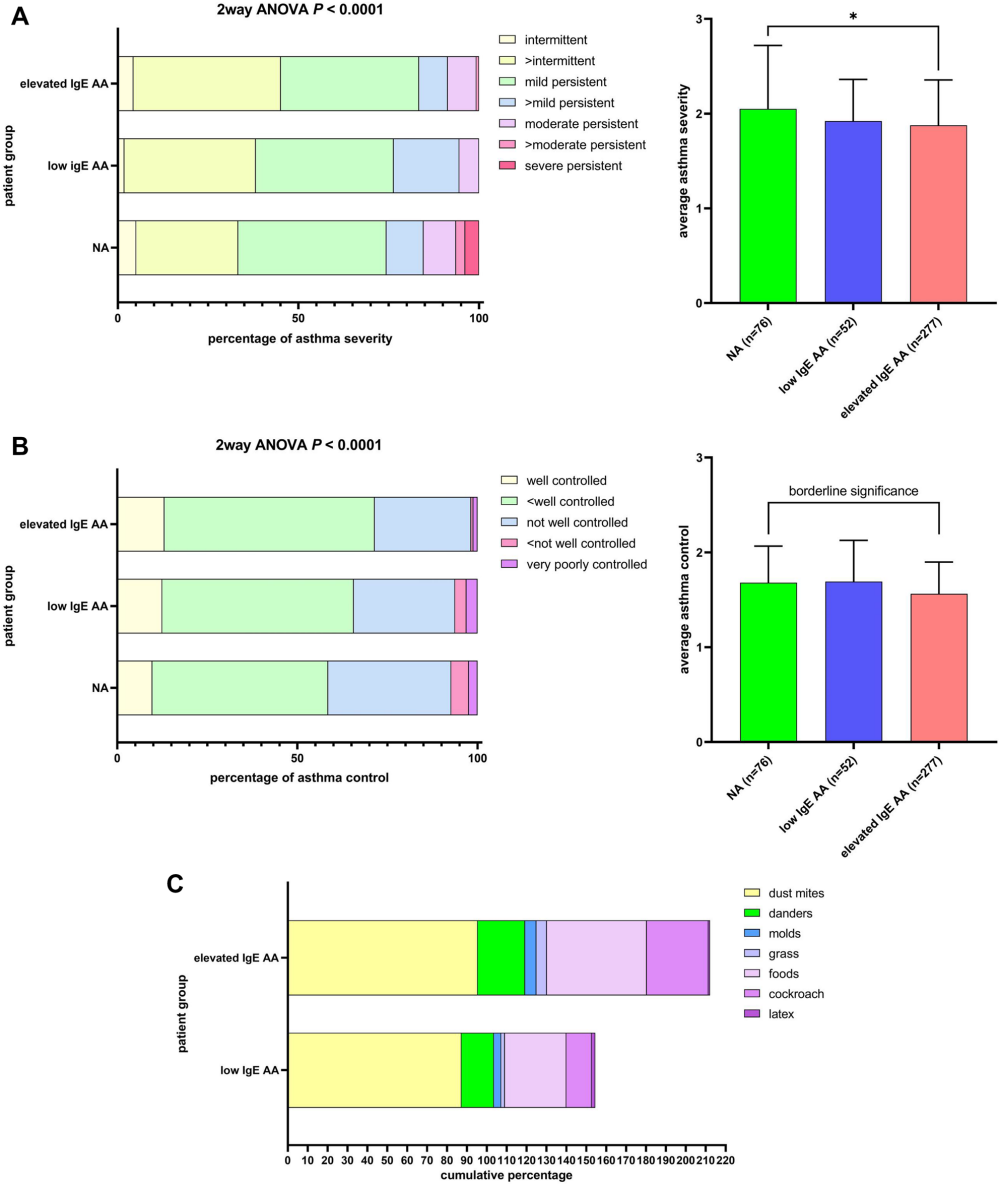

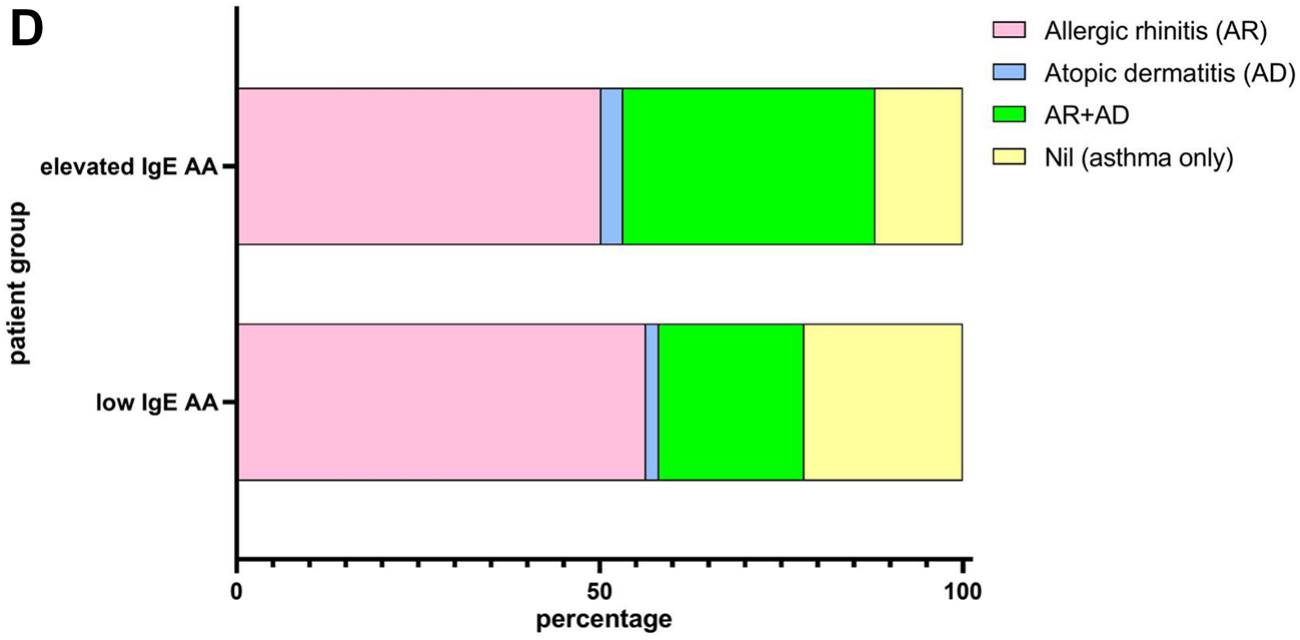
Comparison of asthma severity (**A**), asthma control (**B**), and allergen sensitization (**C**) and allergic comorbidity (**D**) between patients with NA (n = 76), patients with low IgE AA (n = 52), and patients with elevated IgE AA (n = 277). Asthma severity were classified as: intermittent, mild persistent, moderate persistent, and severe persistent. Asthma control were classified as: well controlled, not well controlled and very poorly controlled. *AD* atopic dermatitis, *AR* allergic rhinitis. NA: nonallergic asthma; low IgE AA: allergic asthma with total IgE levels < 150 IU/mL; elevated IgE AA: allergic asthma with total IgE levels > 150 IU/mL.

Higher asthma severity was more frequent in children with NA (41.02%, 8.97%, and 3.85% of those with mild persistent, moderate persistent, and severe persistent asthma) than in those with AA (38.18%, 5.45%, and 0% for patient with low IgE AA and 38.27%, 7.94%, and 0% for patient with elevated IgE AA, respectively) (two-way ANOVA, *P* < 0.0001) (Fig. 1A, left). The average asthma severity of children with NA was significantly higher than that of those with elevated IgE AA (*P* = 0.0104) (Fig. 1A, right). Less controlled asthma was more common in children with NA (34.15%, 4.88%, and 2.44% of those with not well controlled, not well-very poorly controlled, and very poorly controlled asthma) than in children with AA (28.13%, 3.13%, and 3.13% for patient with low IgE AA and 26.79%, 0.60%, and 1.19% for patient with elevated IgE AA, respectively) (two-way ANOVA, *P* < 0.0001) (Fig. 1B, left). The average control level of children with NA was marginally worse than that of those with elevated IgE AA (*P* = 0.0525) (Fig. 1B, right). Mostpatients with elevated IgE AA showed positive sensitization to house dust mite (Fig. 1C). But patient with low IgE AA has a lower rate of allergens sensitization than patient with elevated IgE AA. Most patients with elevated IgE AA had AR and/or AR plus AD (Fig. 1D).

### Asthma severity pattern over time stratifying by the total serum IgE level

**Figure 2:**
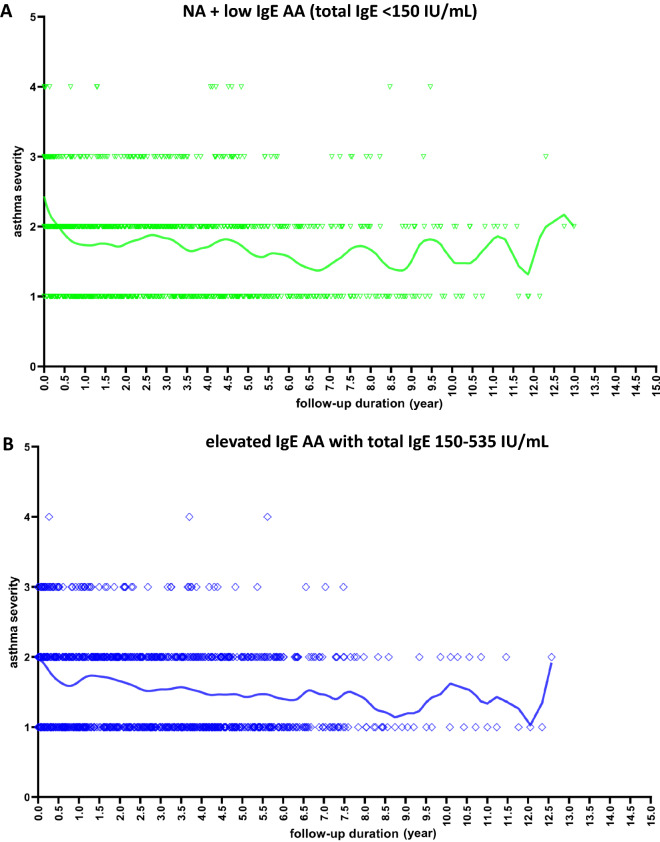

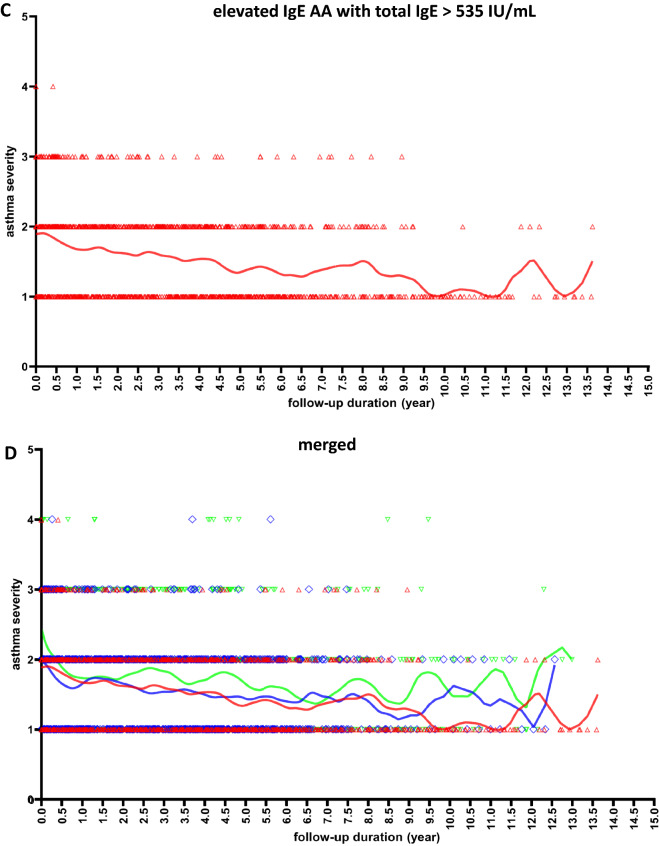
Analysis of the association between follow-up duration and the asthma severity performed by applying a Locally Weighted Scatterplot Smoother (LOWESS) to the patients with total serum IgE < 150 IU/mL (NA + low IgE AA) (**A**; green color, n = 32), elevated IgE AA patients with IgE 150–535 IU/mL (**B**; blue color, n = 32) and elevated IgE AA patients with IgE > 535 IU/mL (**C**; red color, n = 33) and all subjects (**D**; merged, n = 97) by drawing a line through the central tendency of the asthma severity changing with time. The individual data points represent asthma severity at each follow-up visit from each subject. Asthma severity were classified as: intermittent (1 point), mild persistent (2 point), moderate persistent (3 point), and severe persistent (4 point). The fitted line is a LOWESS smoother showing the overall trends of all data point changing with time. NA: nonallergic asthma; low IgE AA: allergic asthma with total IgE levels < 150 IU/mL; elevated IgE AA: allergic asthma with total IgE levels > 150 IU/mL.

Among the patients for whom long-term follow-up data of asthma severity were available, 32 elevated IgE AA patients with total IgE levels 150–535 IU/mL (951 visits) and 33 elevated IgE AA patients with total IgE levels > 535 IU/mL (1154 visits) showed stable patterns or annual improvement of asthma severity over their 10-year follow-ups (Fig. 2). However, among 32 patients with total IgE levels < 150 IU/mL (19 patients with NA + 13 patients with low IgE AA; 1095 visits), the average asthma severity was higher, and intermittent attacks (reflecting increasing severity) persisted. We found that the slopes of NA + low IgE AA (total IgE < 150 IU/mL) group, elevated IgE AA with total IgE 150–535 IU/mL group, and elevated IgE AA with total IgE > 535 IU/mL group are − 0.04378 (95% CI − 0.06034 to − 0.02722), − 0.05871 (95% CI − 0.07575 to − 0.04167), and − 0.07175 (95% CI − 0.08432 to − 0.05919), respectively (Fig. 2). The smaller negative value of the overall slope in the total IgE < 150 IU/mL group means that the improvement of mean asthma severity is less obvious.

### DEmiRNAs in the peripheral blood of patients with NA relative to patients with elevated IgE AA

**Figure 3:**
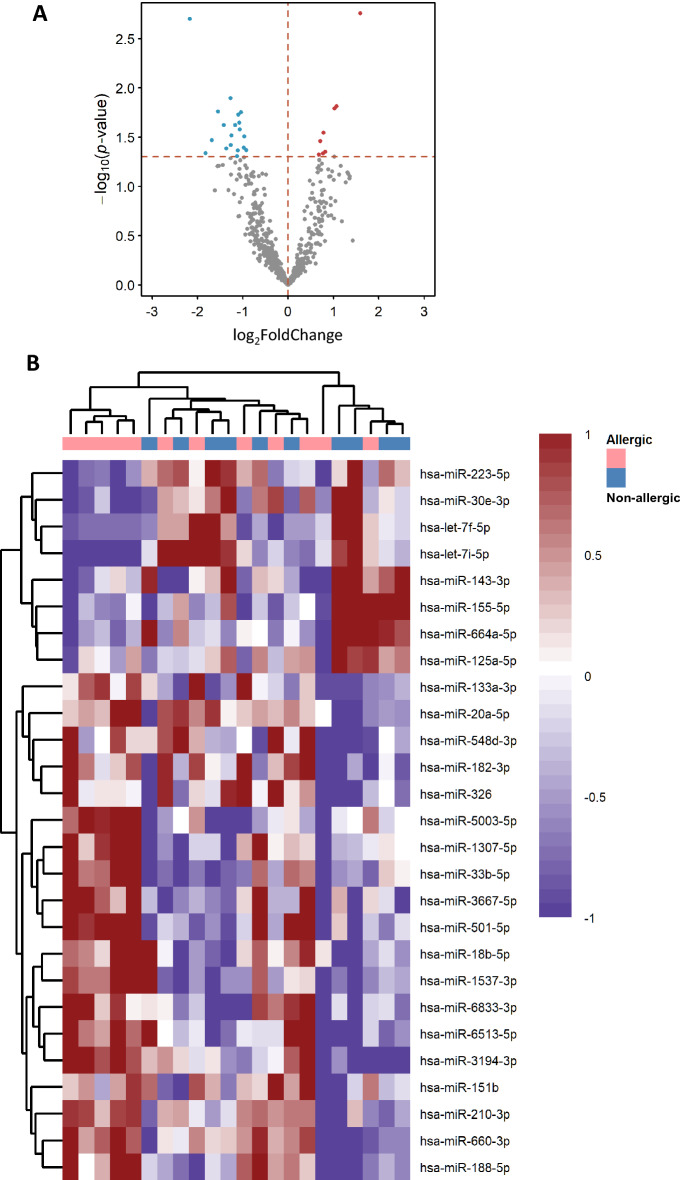
Volcano plot and the heatmap distinguishing nonallergic patients from allergic patients. (**A**) Volcano plot of significant differentially expressed miRNAs (DEmiRNAs) (NA relative to elevated IgE AA) obtained using DESeq2 analysis of RNA-Seq data. The y-axis displays the Log10q-value for each miRNA, while the x-axis displays the Log2FC for that miRNA relative to allergic asthma (nonallergic vs allergic). (**B**) Patterns of expression profiles for the top 27 most highly significantly (*P* < 0.05) DEmiRNAs. The nonallergic patients and allergic patients are represented in columns and the miRNAs are represented in the rows. The color red and blue indicate upregulation and downregulation, respectively. Heatmap was generated by uploading the DEmiRNA data to ClustVis 2.0 (https://biit.cs.ut.ee/clustvis/). NA: nonallergic asthma; elevated IgE AA: allergic asthma with total IgE levels > 150 IU/mL.

We sought to determine whether the expression of miRNAs might be dysregulated in patients with NA compared to elevated IgE AA, therefore we used bulk RNA-seq to compare the whole blood miRNA expression levels in 11 patients with NA and 11 patients with elevated IgE AA. We found 140 DEmiRNAs (based on false discovery rate, FDR: *q-*values < 0.001)^29^ (supplemental Table S2; supplemental Fig. S2). Of these, 68 were upregulated and 72 downregulated. We used the “significance of differential expression” (y-axis: *P* < 0.05) as the cut-off when comparing the log_2_FoldChange (x-axis: log_2_FC). We drew a volcano plot (Fig. 3A) and found 27 DEmiRNAs between patient with NA and patients with elevated IgE AA. Our results exhibited some heterogeneity in patterns of miRNA expression profiles between patient with NA and patients with elevated IgE AA (Fig. 3B).

### KEGG and GO functional pathway analysis based on differentially expressed miRNAs

**Figure 4:**
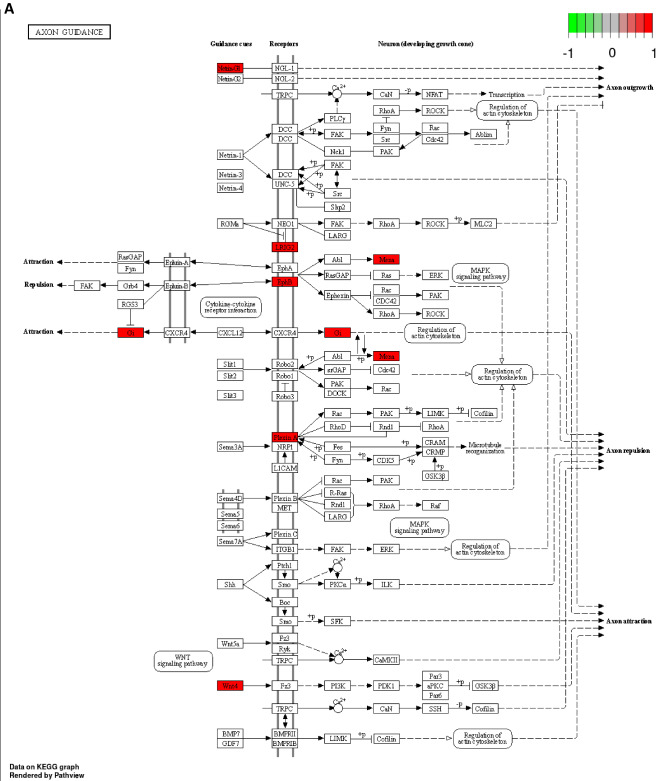

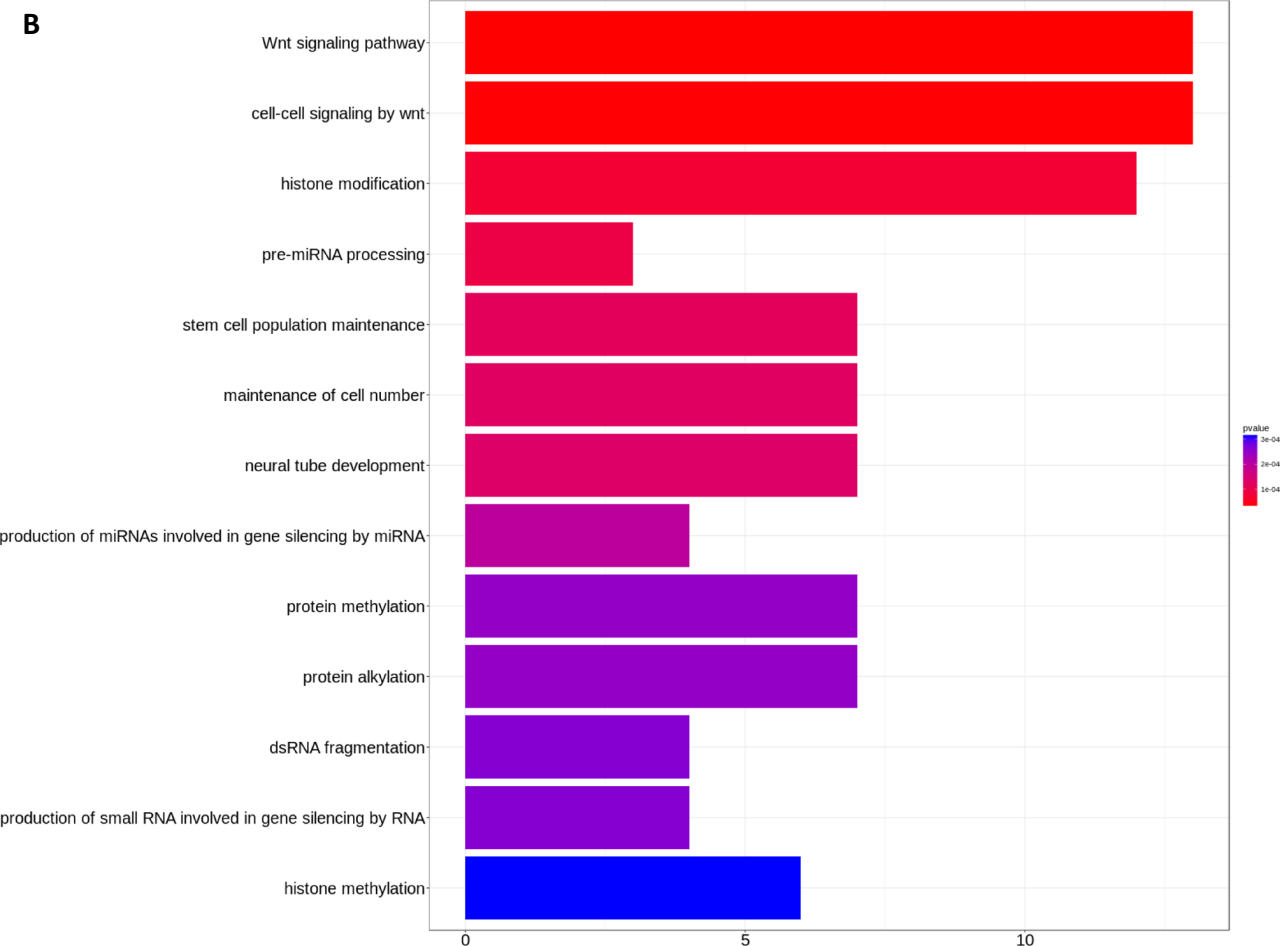
Kyoto Encyclopedia of Genes and Genomes (KEGG) and Gene ontology (GO) analysis based on differentially expressed miRNAs. (**A**) Pathview map of Axon guidance pathway (hsa04360; with permission)^28^. (**B**) Top 13 enriched biological process (BP) were demonstrated. X-axis represent number of predicted target mRNA involved within individual pathway. Red color indicates *P* value of 1 × 10^−4^ while blue color indicates *P* value of 3 × 10^−4^.

The DEmiRNAs between patient with NA and patient with elevated IgE AA were enriched in disorder of axon guidance pathway (hsa04360) recorded in the Kyoto Encyclopedia of Genes and Genomes (KEGG) (Fig. 4A). The top GO enriched biological process were associated with Wnt signaling pathway and histone modification (Fig. 4B).

### Identification of predicted target mRNAs gene associated with airway inflammation in asthma, FcεRI signaling, IL-4 signaling, and the Th2 pathway impacted by DEmiRNAs

**Figure 5:**
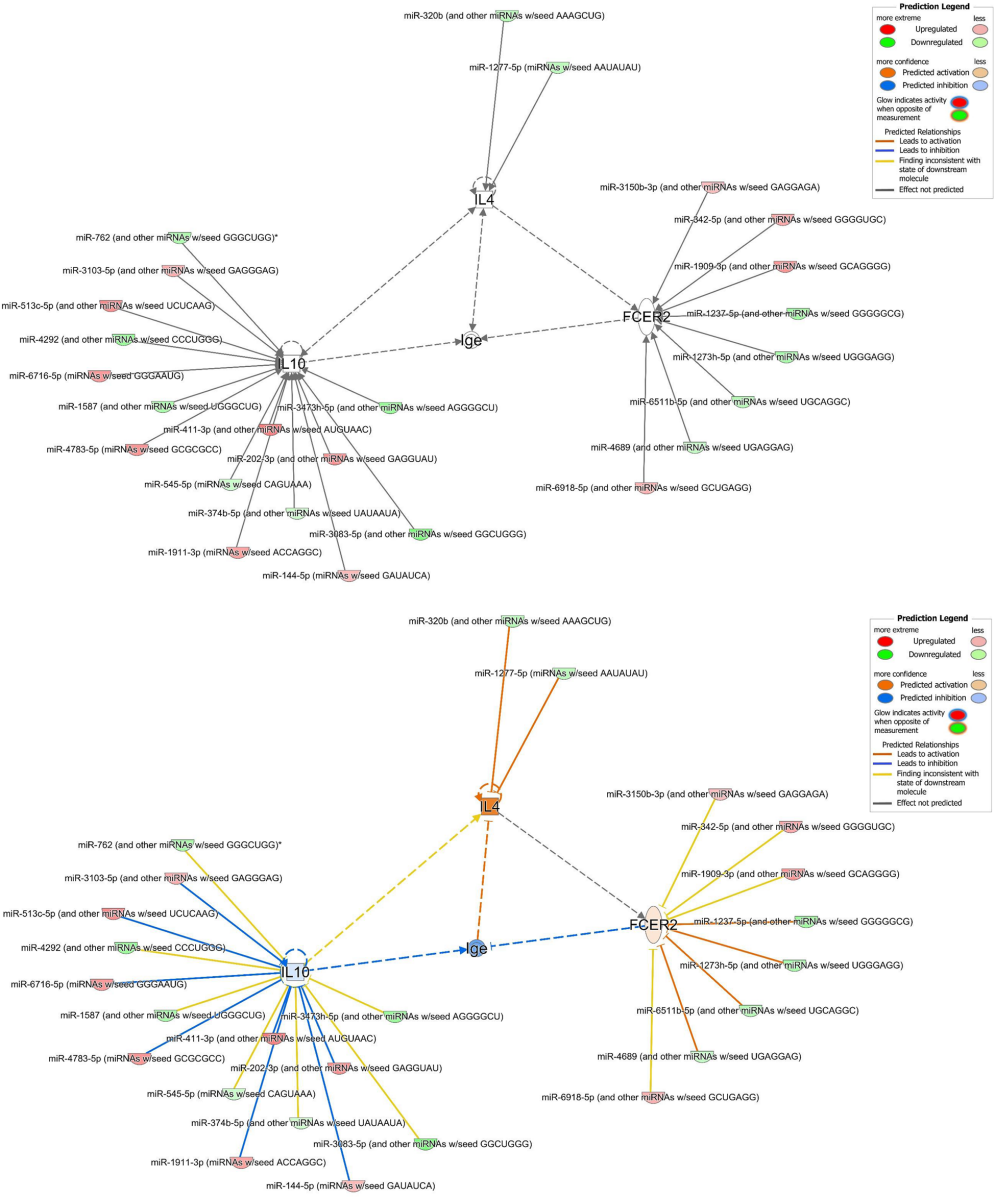

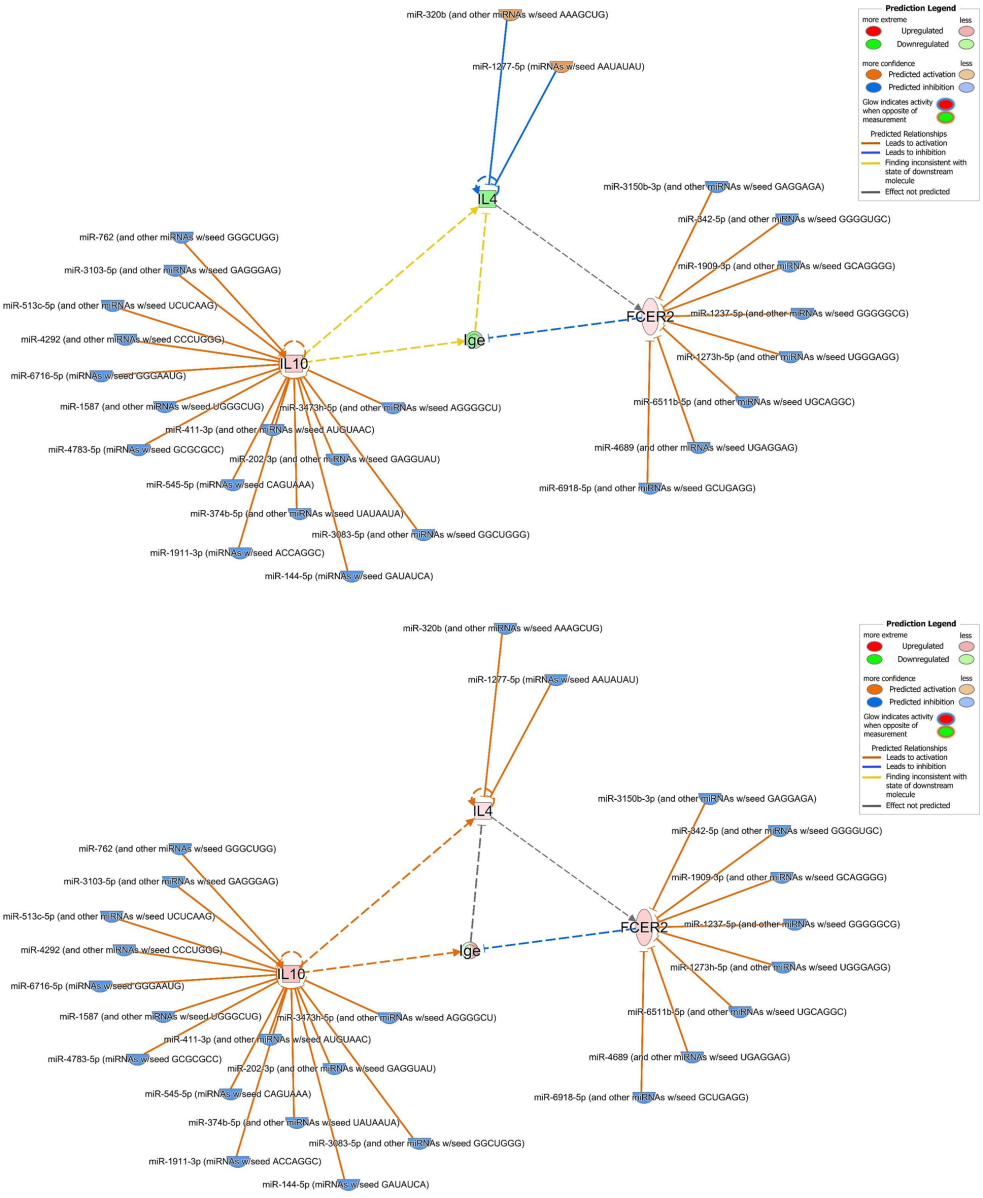
Molecular network linking differentially expressed miRNAs and their predicted target mRNA genes associated with regulation of the expression of IgE. The network displays the predicted target mRNA genes as nodes (vertical oval shape representing transmembrane receptor: *FCER2*; square shape representing cytokine: *IL-4* and *IL-10*) and the biological relationships between the gene nodes and the miRNA node as lines. IgE were linked by *IL-4*, *IL-10*, and *FCER2*, respectively. (**A**) Network whose MAP function had not been turned on. (**B**) Network whose MAP function had been turned on with miRNA data but without any mRNA data overlaid. (**C**) Network whose MAP function had been turned on with mRNA data GSE40887 overlaid. (**D**) Network whose MAP function had been turned on with mRNA data GSE40888 (test 8) overlaid. Full lines represent direct interactions and broken lines, indirect interactions. Orange lines, leads to activation; Blue lines, leads to inhibition; Yellow lines, findings inconsistent with state of downstream molecule; Gray lines, not predicted. The arrowheads indicate the expected effect from the literature, and the edge color signifies the effect the upstream molecule has on the downstream molecule. The color intensity of the mature miRNA node indicates the degree of either upregulation (red) or downregulation (green) of the respective miRNA provided by our miRNA sequencing data. The color intensity of the mRNA gene node indicates the degree of either upregulation (red) or downregulation (green) of the respective mRNA provided by public available mRNA expression data. Where molecules that are predicted to be activated/*increased expression* are colored orange and those predicted to be inhibited/*decreased expression* are colored blue.

We used the *search* and *build-connect* functions of IPA to identify 40 predicted target mRNA genes (Table 2). The analysis networks revealed molecular enriched for or targeted by miRNAs involved in nonallergic childhood asthma. We found that the changes in mRNA expression using publicly available data were evident in some of the predicted target mRNA genes involved in airway inflammation in asthma, FcεRI signaling, IL-4 signaling, and the Th2 pathway (supplemental Figs. S3–S6), in association with altered IgE expression (Fig. 5). We showed genes *IL-4*, *IL-10* and *FCER2* (FcεRII or CD23) were involved in regulating IgE expression (Fig. 5; supplemental Table S3). Two miRNAs, 15 miRNAs, and 8 miRNAs were found to be targeting *IL-4*, *IL-10* and *FCER2*, respectively (Table 3).

**Table 2:** Predicted mRNA target gene of differentially expressed miRNAs.

Symbol	Entrez gene name	Location
*ADRB2*	Adrenoceptor beta 2	Plasma membrane
*ALOX15*	Arachidonate 15-lipoxygenase	Cytoplasm
*BDNF*	Brain derived neurotrophic factor	Extracellular space
*C3AR1*	Complement C3a receptor 1	Plasma membrane
*CCL11*	C–C motif chemokine ligand 11	Extracellular space
*CCL17*	C–C motif chemokine ligand 17	Extracellular space
*CCL5*	C–C motif chemokine ligand 5	Extracellular space
*CD14*	CD14 molecule	Plasma membrane
*CXCL8*	C-X-C motif chemokine ligand 8	Extracellular space
*CYSLTR1*	Cysteinyl leukotriene receptor 1	Plasma membrane
*CYSLTR2*	Cysteinyl leukotriene receptor 2	Plasma membrane
*ERBB2*	erb-b2 receptor tyrosine kinase 2	Plasma membrane
*FCER2*	Fc fragment of IgE receptor II	Plasma membrane
*HAVCR2*	Hepatitis A virus cellular receptor 2	Plasma membrane
*IgE*	Immunoglobulin E	Extracellular space
*IL10*	Interleukin 10	Extracellular space
*IL13*	Interleukin 13	Extracellular space
*IL1A*	Interleukin 1 alpha	Extracellular space
*IL1B*	Interleukin 1 beta	Extracellular space
*IL1RAP*	Interleukin 1 receptor accessory protein	Plasma membrane
*IL1RL1*	Interleukin 1 receptor like 1	Plasma membrane
*IL2*	Interleukin 2	Extracellular space
*IL3*	Interleukin 3	Extracellular space
*IL33*	Interleukin 33	Extracellular space
*IL4*	Interleukin 4	Extracellular space
*IL4R*	Interleukin 4 receptor	Plasma membrane
*IL7R*	Interleukin 7 receptor	Plasma membrane
*ITGB3*	Integrin subunit beta 3	Plasma membrane
*MMP9*	Matrix metallopeptidase 9	Extracellular space
*MS4A2*	Membrane spanning 4-domains A2	Plasma membrane
*NGF*	Nerve growth factor	Extracellular space
*NOS2*	Nitric oxide synthase 2	Cytoplasm
*NRG1*	Neuregulin 1	Plasma membrane
*NTRK2*	Neurotrophic receptor tyrosine kinase 2	Plasma membrane
*PDE4D*	Phosphodiesterase 4D	Cytoplasm
*STAT6*	Signal transducer and activator of transcription 6	Nucleus
*TGFB1*	Transforming growth factor beta 1	Extracellular space
*TLR4*	Toll like receptor 4	Plasma membrane
*TNFSF4*	TNF superfamily member 4	Extracellular space
*TSLP*	Thymic stromal lymphopoietin	Extracellular space

**Table 3:** MicroRNAs and their predicted target mRNAs involved in regulating expression of IgE.

From molecule(s)	Relationship type	To molecule(s)
miR-1237-5p (and other miRNAs w/seed GGGGGCG)	microRNA targeting	*FCER2*
miR-1273 h-5p (and other miRNAs w/seed UGGGAGG)	microRNA targeting	*FCER2*
miR-1277-5p (miRNAs w/seed AAUAUAU)	microRNA targeting	*IL4*
miR-144-5p (miRNAs w/seed GAUAUCA)	microRNA targeting	*IL10*
miR-1587 (and other miRNAs w/seed UGGGCUG)	microRNA targeting	*IL10*
miR-1909-3p (and other miRNAs w/seed GCAGGGG)	microRNA targeting	*FCER2*
miR-1911-3p (miRNAs w/seed ACCAGGC)	microRNA targeting	*IL10*
miR-202-3p (and other miRNAs w/seed GAGGUAU)	microRNA targeting	*IL10*
miR-3083-5p (and other miRNAs w/seed GGCUGGG)	microRNA targeting	*IL10*
miR-3103-5p (and other miRNAs w/seed GAGGGAG)	microRNA targeting	*IL10*
miR-3150b-3p (and other miRNAs w/seed GAGGAGA)	microRNA targeting	*FCER2*
miR-320b (and other miRNAs w/seed AAAGCUG)	microRNA targeting	*IL4*
miR-342-5p (and other miRNAs w/seed GGGGUGC)	microRNA targeting	*FCER2*
miR-3473 h-5p (and other miRNAs w/seed AGGGGCU)	microRNA targeting	*IL10*
miR-374b-5p (and other miRNAs w/seed UAUAAUA)	microRNA targeting	*IL10*
miR-411-3p (and other miRNAs w/seed AUGUAAC)	microRNA targeting	*IL10*
miR-4292 (and other miRNAs w/seed CCCUGGG)	microRNA targeting	*IL10*
miR-4689 (and other miRNAs w/seed UGAGGAG)	microRNA targeting	*FCER2*
miR-4783-5p (miRNAs w/seed GCGCGCC)	microRNA targeting	*IL10*
miR-513c-5p (and other miRNAs w/seed UCUCAAG)	microRNA targeting	*IL10*
miR-545-5p (miRNAs w/seed CAGUAAA)	microRNA targeting	*IL10*
miR-6511b-5p (and other miRNAs w/seed UGCAGGC)	microRNA targeting	*FCER2*
miR-6716-5p (miRNAs w/seed GGGAAUG)	microRNA targeting	*IL10*
miR-6918-5p (and other miRNAs w/seed GCUGAGG)	microRNA targeting	*FCER2*
miR-762 (and other miRNAs w/seed GGGCUGG)	microRNA targeting	*IL10*

Figure 5A showed the molecular network and the miRNA expression based on our sequencing measurement data. MAP has not been used to predict the overall effects of interactions between molecules in a network. Figure 5B: through the prediction of MAP, it is shown that miRNAs’ negative regulatory effect on the expression of protein-coding genes. We showed that *IL-4* and *FCER2* were predicted to be activated, whereas *IL-10* was predicted to be downregulated. IgE expression was predicted to be inhibited under interaction with *IL-4*, activation of *IL-10*, and inhibition of *FCER2*. Figure 5C: through the prediction of MAP, it is shown that the regulatory effect of public mRNA data on expression of IgE. We showed that, on addition of the publicly available mRNA data from CD4^+^ T cells of children with AA and with NA (GSE40887), *IL-10* and *FCER2* were upregulated when miRNAs were predicted to be downregulated. *IL-4* was downregulated when miRNAs were predicted to be upregulated. Notably, IgE expression was downregulated due to the inhibitory effect from *FCER2*. Figure 5D: through the prediction of MAP, it is shown that the different regulatory effect of another public mRNA data affects the expression of IgE. We showed that when publicly available mRNA data from PBMCs of children with AA and with NA (GSE40888) were added, *IL-4*, *IL-10,* and *FCER2* were upregulated when miRNAs were predicted to be downregulated. Interestingly, unlike Fig. 5C, because there were simultaneous an inhibitory effect from FCER2 and a activating effect from IL-10, the expression of IgE may be downregulated or upregulated.

Whether we used our miRNA measurement data to make predictions, or we used two publicly available mRNA expression data to make predictions, the expression level of IgE was downregulated. This means that the molecular network we constructed using DEmiRNAs data could predict NA related miRNA regulation and miRNA-predicted target mRNA interactions.

## Discussion

### Childhood asthma phenotype stability

Asthma in children is a chronic inflammatory disease now considered a broad spectrum of diseases with distinct phenotypes (variable clinical presentations) and endotypes (characteristic mechanistic pathways). A considerable problem in many studies that attempt to determine phenotypes outcomes in childhood asthma is that we only assess single episodes or acute symptoms that occurred over a short period of time but we do not take into account the long-term stability of asthma phenotypes outcomes^30^. We showed the average asthma severity was higher, and intermittent attacks (reflecting increasing severity) persisted for nonallergic childhood asthma in our follow-up study of duration 5–15 years. In line with Siroux et al*.*, we found that NA in children was more severe than AA^31^. The fact that a majority of subjects with a specific phenotype remained in the same phenotype over a 10-year observation period (54–88%)^32^ suggests that asthma phenotypes remain stable^22^. This point-of-view might explain that the same phenotype in our study includes children of different age ranges.

### Nonallergic phenotype in childhood age

The nonallergic form predominates among older patients (typically seen in those aged > 30 years), with a female predominance^9–11^. Unlike most previous studies, Deliu et al*.* identified an early onset non-atopic asthma subtype with an average age of onset of 3.8 years (range 2–6 years)^33^. Nonallergic asthma is most common in patients ages < 4 years or > 40 years^34^. We found that the mean age of patients with NA at enrolment was 5.614 ± 2.743 years. We identified and confirmed that the nonallergic phenotype of childhood asthma at a younger age. While we did not find a significant association between age at enrollment and average asthma severity in patients with NA, there was a weak negative correlation observed. It is possible that higher asthma severity in NA might contribute to earlier age among NA patients, but further research is needed to confirm this.

### Low IgE and negative sensitization in nonallergic asthmatics

The generally accepted definition of NA includes asthma without allergic sensitization to a panel of allergens in skin prick or in vitro allergen-specific IgE tests and that the total serum IgE level is typically normal or low^11,12,22^. We demonstrated that the total serum IgE levels of both children with NA and children with low IgE AA was significantly lower than that of those with elevated IgE AA. The dominant clinical phenotype in severe asthma tends to be NA, with IgE levels lower than those seen in subjects with mild-to-moderate AA^35^. However, little is known concerning the cause of low level of total serum IgE and negative allergic sensitization in skin prick or in vitro allergen-specific IgE tests. We found the expression level of predicted target mRNA genes related to airway inflammation in asthma, FcεRI signaling, IL-4 signaling, and Th2 pathway were modified. Certain factors explain the different IgE reactivity between patient with AA and patients with NA^11^. Virchow et al*.* reported that negative sensitization has been attributed to low total IgE levels in patients with NA^36^. Tsolakis et al*.* confirmed total IgE was 21.8 IU/mL (16.3–29.1 IU/mL) for patient with allergen-specific IgE < 0.35 IU/mL while total IgE was 12.4 kU/L (8.53–18.2 IU/mL) for patient with allergen-specific IgE < 0.10 IU/mL^37^. We showed that patient with low IgE AA (IgE < 150 IU/mL) has a lower rate of allergens sensitization than patient with elevated IgE AA. Collectively, these evidence indicated that presence of allergen-specific IgE (sensitization) was highly associated with total serum IgE levels in patients with NA. We speculate that miRNA in some extent modify (or suppress) IgE expression and Th2 allergic inflammation which explained low IgE level and negative allergen-specific IgE tests in NA.miRNA and predicted target mRNA interactions.

The functional roles of miRNAs in patients with NA remain poorly understood. However, some miRNAs we identified have been reported to be linked to asthma or allergies. In asthmatics, the expression of miR-1237-5p was upregulated in airway epithelial cell lines^38^. MiR-1273 h-5p expression was significantly elevated in status asthmaticus^39^. MiR-144-5p was upregulated in asthma patients and considered to serve as a diagnostic biomarker^40,41^. The expression of miR-1587 is upregulated while that of miR-374b-5p were downregulated in children with asthma exacerbation^42^. The reduction in miR-202-3p expression correlated with a reduction in predicted FEV_1_ percentage^43^. It has been suggested that miR-320 exhibits anti-inflammatory effects and was associated with asthma remission^44^. MiR-342-5p is coupled to the antiviral interferon (IFN) response via IFN regulatory factor 1 (IRF1) and plays roles in both asthma and respiratory infections^45^. MiR-513c-5p decreases the secretion of Th1 cytokines^46^.

We identified 25 miRNAs that regulated the expression of *IL-4* (2 miRNAs), *IL-10* (15 miRNAs), and *FCER2* (8 miRNAs) (Table 3, Supplemental Table S2). Baos et al. reported that *IL-10* could be used to discriminate AA from NA, and that *IL-10* levels were higher in patients with severe asthma^10^. Both T-reg and B-reg cells suppress IgE production and induce the synthesis of IgG4-isotype allergen-specific antibodies (particularly via IL-10 activity)^47^. IL-10 could act indirectly through accessory cells present in PBMCs to decrease IgE production^48^. Higher *FCER2* (CD23) expression on monocytes and higher sCD23 levels were observed to result in decreasing IgE production in intrinsic asthmatic patients^49^. *FCER2* (CD23) is also involved in feedback regulation of IgE production^50^. We found that *FCER2* expression always inhibited IgE synthesis; the IgE level was affected by the expression of both *IL-4* and *IL-10*. When the expression level of *IL-4* gene is downregulated, the expression level of IgE is also downregulated at this time. Collectively, the DEmiRNAs we identified, and their predicted target mRNAs network, might associate with the low total IgE and negative sensitization in nonallergic childhood asthma. Our observations on the association of multiple miRNAs and their predicted target mRNAs in nonallergic childhood asthma highlight their effect on the control of inflammatory responses, Th2 polarization, and disease severity, which can potentially allow delivery of precision medicine to childhood asthma.

Our KEGG and GO Functional Pathway Analysis also implied both low IgE response and asthma persistence in nonallergic childhood asthma. Dysregulated Wnt signaling has been linked to the pathogenesis of airway remodeling in asthmatic patients. Recent evidences have shown that activation of the Wnt-1-driven canonical Wnt signaling pathway resulted in suppression of both airway inflammation and airway inflammation^51^. In a study of children with mild to moderate persistent asthma, polymorphisms in genes encoding WNT-1-inducible signaling pathway protein 1 (WISP-1) and WNT inhibitory factor-1 (WIF-1), respectively, are associated with impaired lung function in childhood asthma^52^. There is a deranged balance between Wnt enhancer and Wnt inhibitors in the bronchial epithelium of severe asthmatic patients. The Th2-high asthma phenotype is associated with upregulated Wnt-negative regulators, while higher canonical Wnt signaling enriched pathway are observed in both inflammatory and severe neutrophilic asthmatics^53^.

### Inhaled corticosteroid (ICS)

Hsa04360 pathway had been reported to be associated with inhaled corticosteroid (ICS) treatment and glucocorticoid receptor (GR) isoform β (GRβ) overexpression in HeLa cells^54,55^. In a study concerning regulation of airway smooth muscle proliferation, Fluticasone did not increase the enrichment of hsa04360: axon guidance related biological processes^56^. We speculated that hsa04360: axon guidance biological processes were associated with ICS responsiveness and also with airway remodeling.

In both Table 1 and Supplement Table S1, we showed that the ICS dose of NA group was higher than the other two AA groups. Weidner et al. studied circulating miRNA (miR-126, miR-145, miR-146a, miR-155, miR-223, and miR-374a. They reported that miR-146a and miR-155) expression in serum samples of individuals with allergic asthma (AA) and non-allergic asthma (NAA). They found that miR-146a and miR-155 were increased in AA subjects using ICS, but no significant changes were observed in NAA subjects^57^. The altered miRNAs in this study were not in our top 140 list of DEmiRNAs. Liang et al. reported ICS/LABAs treatment reduced airway inflammation and remodeling, but did not completely suppress allergic stimulation related pathways in treated patients. IgE levels after treatment also showed a decreasing trend, but without statistical significance^58^. However, our target predicted mRNA, IL-4, IL-10, and FCER2, were not reported in this RNA transcription study. ICS treatment's impact on inflammatory phenotype has been inconsistent, and there is limited data on its effects on children. Although there is variability in inflammatory phenotypes, there is no indication that this correlates with changes in ICS dosage^59^. Our KEGG analysis showed that DEmiRNAs in normal and allergic asthma patients were enriched in the axon guidance pathway (hsa04360) (Fig. 4A), which we suggest to be associated with ICS responsiveness and airway remodeling. However, a study on airway smooth muscle proliferation found no increase in axon guidance pathway (hsa04360) with ICS fluticasone treatment^56^. The main functional effect of ICS after inhalation into the airways is lung retention and ICS get rapid systemic breakdown at the same time. Based on these evidences mentioned above, it is inconclusive whether low-medium ICS in our study might have an impact on the miRNA transcriptome analysis of blood cells related to total IgE associated airway inflammation.

## Conclusion

**Figure 6:**
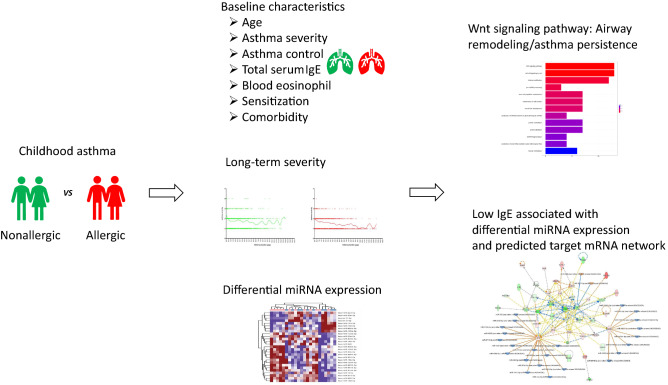
Overview of experimental workflow, study design, and analysis of differentially expressed miRNA data.

The generally accepted definition of NA is asthma without allergic sensitization and the total serum IgE level is typically normal or low. We speculate that NA might involve an inflammatory pathway that in some extent differs from that of Th2 allergic inflammation. Our major findings were depicted schematically in Fig. 6. The comparison between nonallergic and allergic childhood asthma showed that some of the clinical characteristics of patients with NA are distinct from those of patients with elevated IgE AA. Our heat map results exhibited some heterogeneity in patterns of miRNA expression profiles between patients with NA and patients with elevated IgE AA. DEmiRNAs signatures associate with downregulation of total IgE expression and predicted target mRNA genes related molecular networks contribute to canonical pathways of nonallergic childhood asthma. We demonstrated certain factors (DEmiRNAs) explain the different IgE response between patients with NA and patients with elevated IgE AA.

### Limitations

Our miRNA data is obtained only from 11 patients within each asthma type, and there may be selection biases because of low case number. Our evidence real-world studies are useful in that which mirrors what occurs in clinical practice. For the expression of predicted target mRNA gene, further experiments are necessary to verify the real expression of target mRNA genes or proteins. There are many other molecules within the molecular network (including miRNA and mRNA) that we have constructed, and they are very likely to be involved in the regulation of nonallergic inflammation. We use IPA database to find molecular network or functional pathway related to NA, so it is possible that we are unable to find all navel molecular network or functional pathways based on the existing known evidence.

## Supplementary Information

Supplementary Information 1.

Supplementary Information 2.

Supplementary Information 3.

Supplementary Information 4.

Supplementary Information 5.

Supplementary Information 6.

Supplementary Information 7.

Supplementary Information 8.

Supplementary Information 9.

## Data Availability

All data are available in the manuscript and as supplement online. The datasets generated and/or analyzed during the current study are available in the NTU Space. [https://www.space.ntu.edu.tw/navigate/s/6613371C3DD7417B9B84E784819FB659QQY] and Gene Expression Omnibus (GEO) series accession number, GSE222775.

## Abbreviations

AR: Allergic rhinitis
AD: Atopic dermatitis
DEmiRNA: Differentially expressed miRNA
ICS: Inhaled corticosteroid
mRNA: Messenger RNA
miRNA: MicroRNA
LOWESS: Locally weighted scatterplot smoothing
STAT-6: Signal transducer and activator of transcription 6
Th2: T helper cell type 2
ILC2: Type 2 innate lymphoid cells
PBMC: Peripheral blood mononuclear cell
RPM: Reads per million
