# Supplementary material for: Differentially expressed microRNAs in peripheral blood cell are associated with downregulated expression of IgE in nonallergic childhood asthma
Source: Sci Rep. 2023 Apr 19;13:6381. doi: 10.1038/s41598-023-33663-5 (PMC10115804; doi:10.1038/s41598-023-33663-5)

**Supplement Figure 1.** Comparison of age (A), total serum IgE (B) and blood eosinophil percentage (C) between NA, low IgE AA, and elevated IgE AA. Vertical bars represent standard error of the mean. *: *P*<0.05; **: *P*<0.01; ***: *P*<0.001; ****: *P*<0.0001. NA: nonallergic asthma; low IgE AA: allergic asthma with total IgE levels < 150 IU/mL; elevated IgE AA: allergic asthma with total IgE levels > 150 IU/mL.


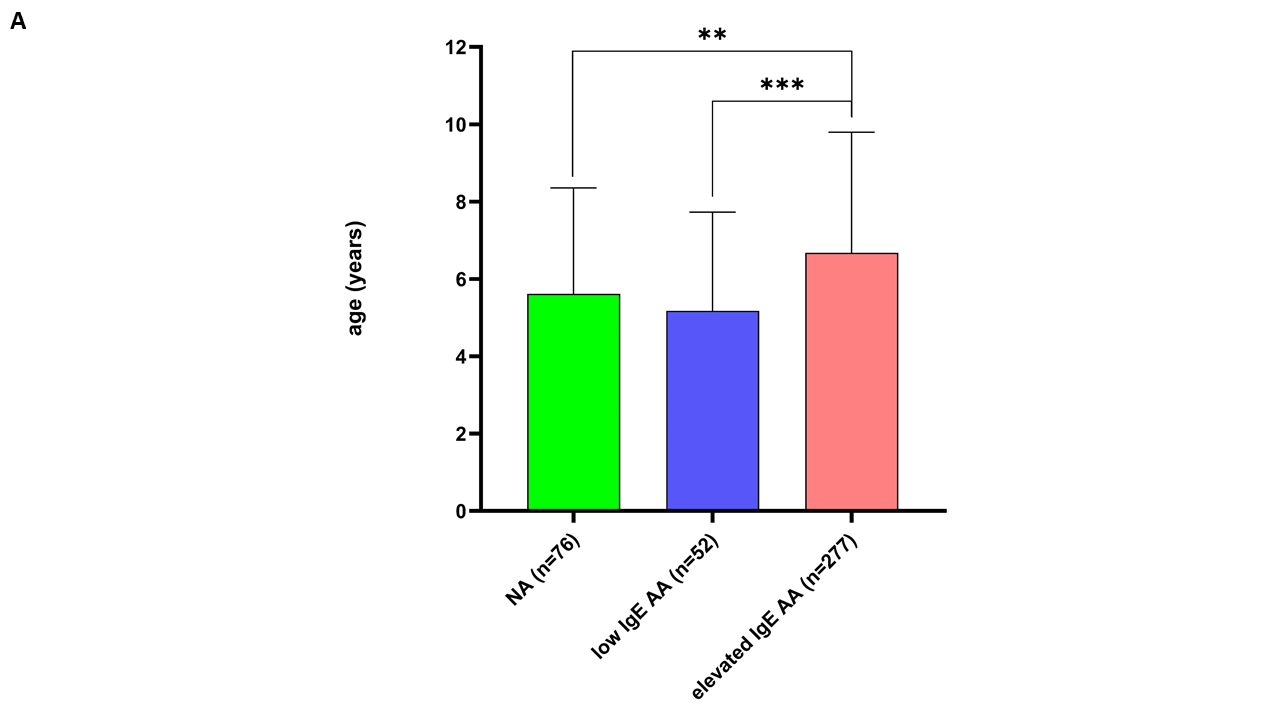


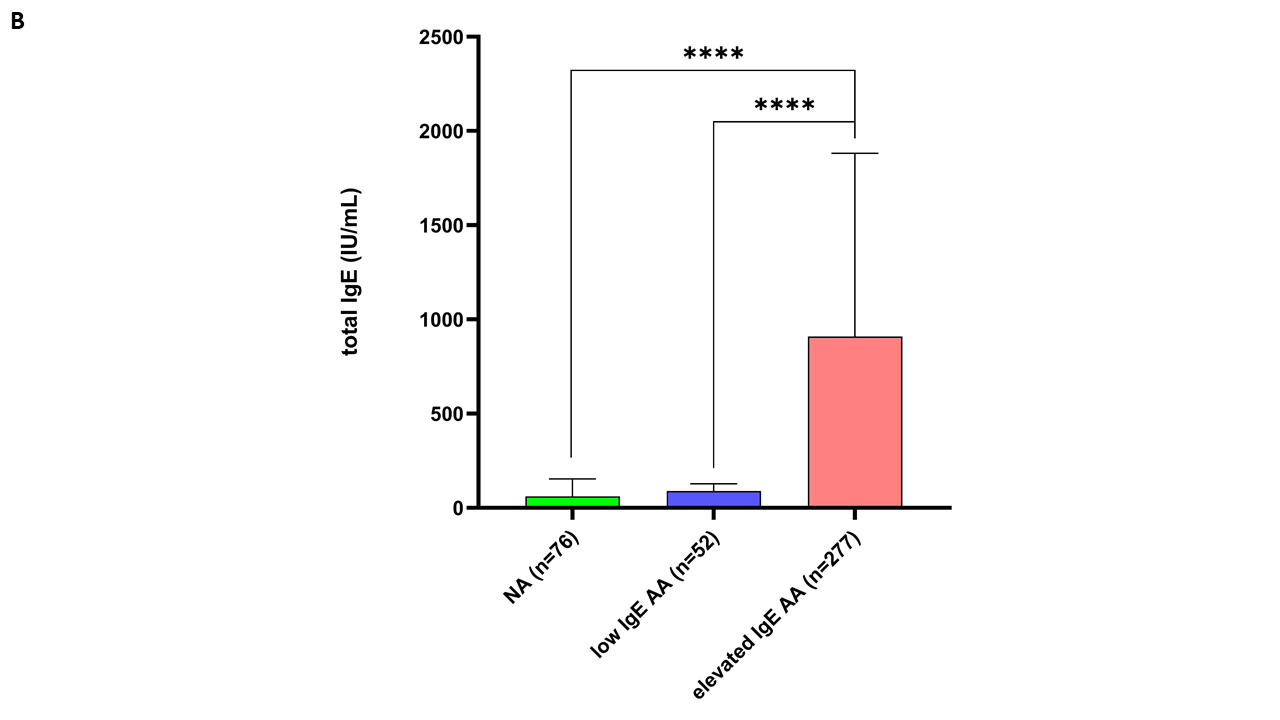


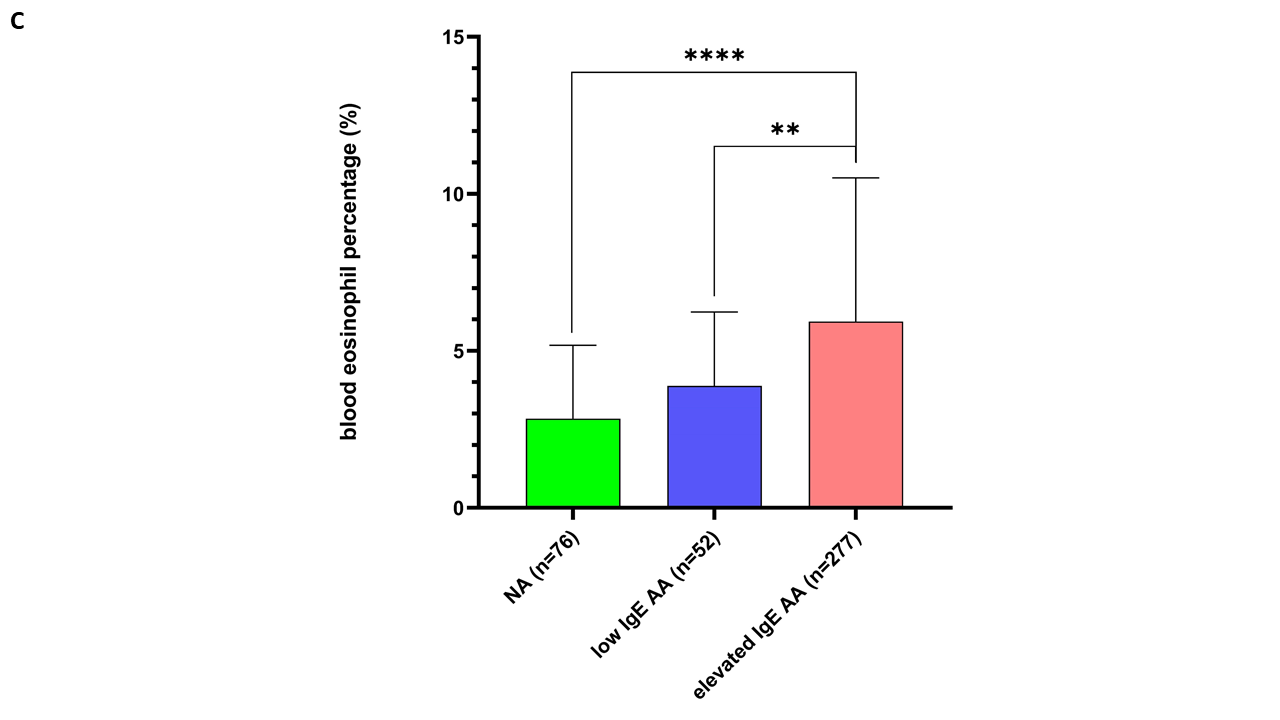

Supplement: Supplementary file 1 — Supplementary Information 1. [file 41598_2023_33663_MOESM1_ESM.docx]
