# Supplementary figures and images for: Differentially expressed microRNAs in peripheral blood cell are associated with downregulated expression of IgE in nonallergic childhood asthma
Source: Sci Rep. 2023 Apr 19;13:6381. doi: 10.1038/s41598-023-33663-5 (PMC10115804; doi:10.1038/s41598-023-33663-5)

**Supplement Figure 2.** Heatmap of 140 top differentially expressed miRNAs.


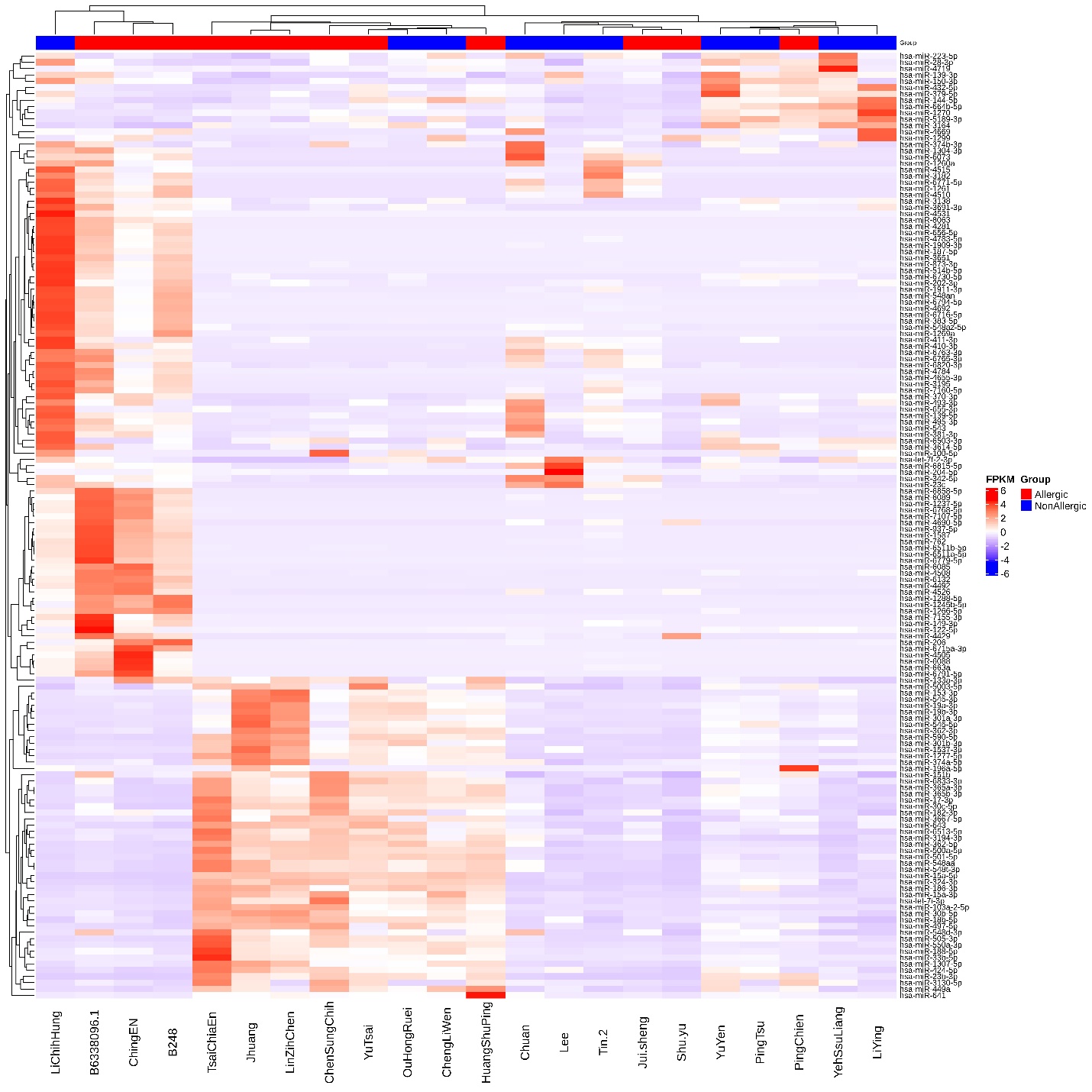

Supplement: Supplementary file 2 — Supplementary Information 2. [file 41598_2023_33663_MOESM2_ESM.docx]
