# Supplementary material for: Differentially expressed microRNAs in peripheral blood cell are associated with downregulated expression of IgE in nonallergic childhood asthma
Source: Sci Rep. 2023 Apr 19;13:6381. doi: 10.1038/s41598-023-33663-5 (PMC10115804; doi:10.1038/s41598-023-33663-5)
Supplement: Supplementary file 3 — Supplementary Information 3. [file 41598_2023_33663_MOESM3_ESM.docx]

**Supplement Figure 3.** Canonical Pathway of airway inflammation in asthma. (A) Application with Publicly available RNA-seq data published under the accession GSE40887. (B) Application with Publicly available RNA-seq data published under the accession GSE40888 (test 8). Red represent upregulated; Green represent downregulated.

**
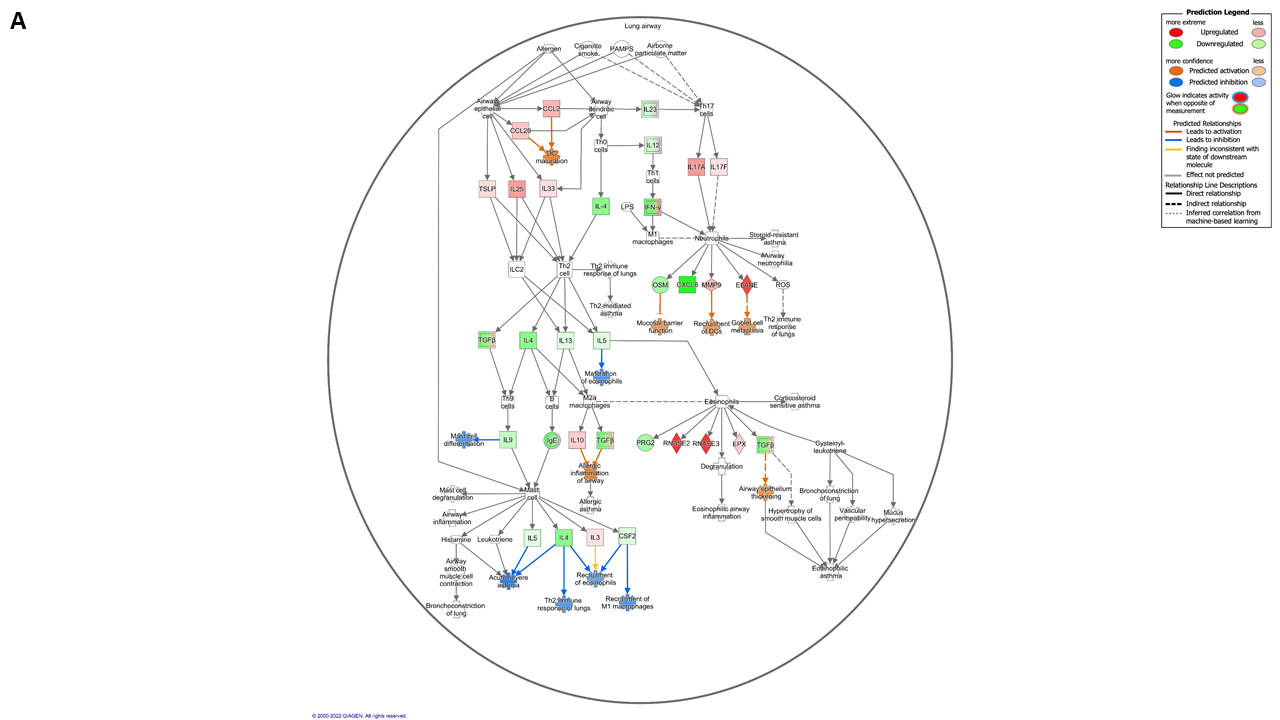
**

**
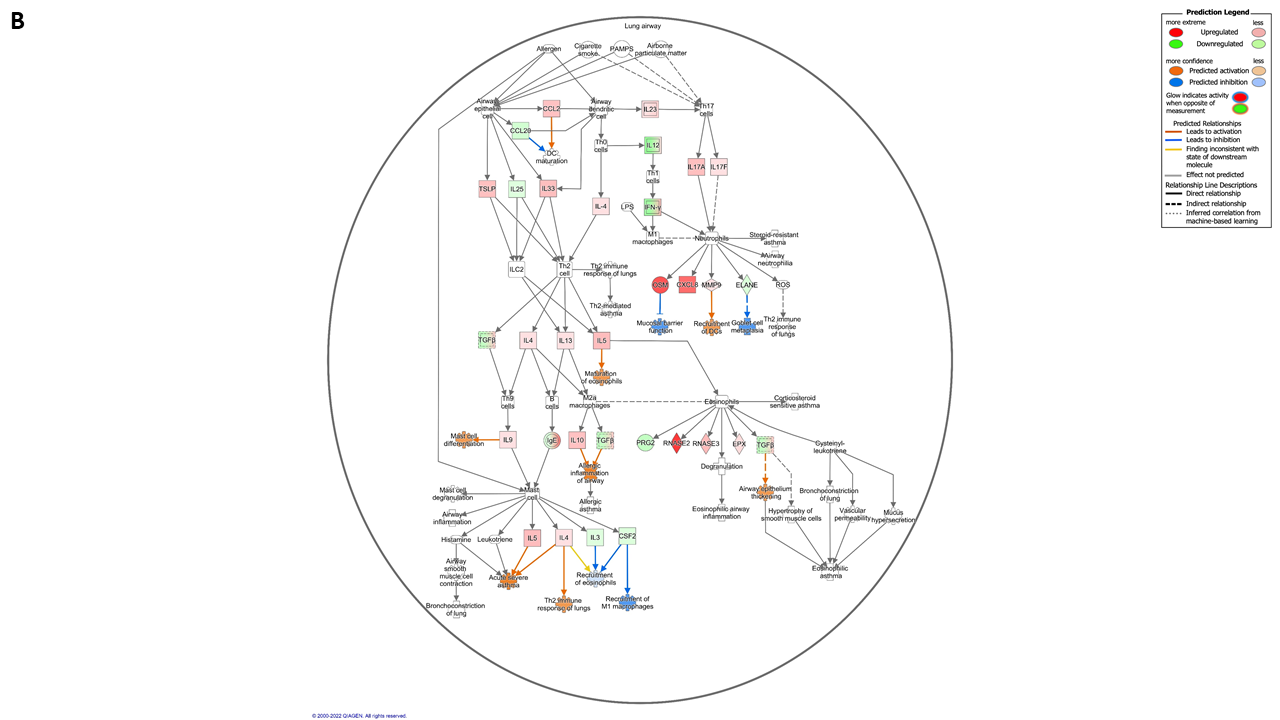
**
