# Supplementary material for: Differentially expressed microRNAs in peripheral blood cell are associated with downregulated expression of IgE in nonallergic childhood asthma
Source: Sci Rep. 2023 Apr 19;13:6381. doi: 10.1038/s41598-023-33663-5 (PMC10115804; doi:10.1038/s41598-023-33663-5)
Supplement: Supplementary file 7 — Supplementary Information 7. [file 41598_2023_33663_MOESM7_ESM.docx]

**Supplement Table 1.** Characteristics of 22 subjects with miRNA analysis

|  | Subjects with  NA (n=11) | Subjects with  elevated IgE AA (n=11) |
| --- | --- | --- |
| Age at enrollment:  years, mean±SD (range) | 7.15014  (2-12.1875) | 9.78485  (2-17) |
| F:M (ratio) | 5:6 | 4:7 |
| Average asthma severity (mean±SD ) † | 3.27273±0.564076 | 2.45455±0.650175 |
| Total serum IgE  (IU/mL; mean±SD)  (range) | 48.9864±36.10782  (5.27-115) | 737.818±430.4943  (318-1707) |
| Peripheral blood eosinophil (%; mean±SD) (range) | 2.55455±1.647643  (0.1-5.0) | 5.32727±4.027677  (1.2-14.0) |
| Sensitization status (%) |  |  |
| dust mites | 0 | 90.9 |
| danders | 0 | 9.09 |
| molds | 0 | 0 |
| grass | 0 | 9.09 |
| foods | 0 | 27.27 |
| cockroach | 0 | 27.27 |
| latex | 0 | 0 |
| Inhaled corticosteroids  (ICS) dose (μg/day)^Ψ^ | 228.5 ± 88.8 | 194.7 ± 88.1 |

Ψ: Fluticasone propionate equivalent according to GINA 2019, Box 3-6.
