# Supplementary material for: Differentially expressed microRNAs in peripheral blood cell are associated with downregulated expression of IgE in nonallergic childhood asthma
Source: Sci Rep. 2023 Apr 19;13:6381. doi: 10.1038/s41598-023-33663-5 (PMC10115804; doi:10.1038/s41598-023-33663-5)
Supplement: Supplementary file 8 — Supplementary Information 8. [file 41598_2023_33663_MOESM8_ESM.docx]

**Supplement Table 2.** List of top 140 differentiated expressed microRNA between nonallergic and allergic asthma patients.

**68 Upregulated microRNA**

| **nonallergic Expression Intensity: FPKM** | **allergic Expression Intensity: FPKM** | **Log_2_FoldChange normalized** | **z-score** | **p-value** | **Expression False Discovery Rate (q-value)** | **Symbol** |
| --- | --- | --- | --- | --- | --- | --- |
| 19.668 | 1.833 | 3.391 | -4.073 | 0.0000465 | 0.000233 | miR-4515 (miRNAs w/seed GGACUGG) |
| 25.692 | 3.521 | 2.835 | -4.285 | 0.0000183 | 0.0000947 | miR-374c-5p (and other miRNAs w/seed UAAUACA) |
| 23.167 | 3.583 | 2.66 | -3.936 | 0.000083 | 0.000407 | miR-4531 (miRNAs w/seed UGGAGAA) |
| 38.5 | 6.225 | 2.596 | -5.007 | 5.53E-07 | 0.00000322 | miR-204-5p (and other miRNAs w/seed UCCCUUU) |
| 33.974 | 7.668 | 2.115 | -4.165 | 0.0000312 | 0.000159 | miR-411-3p (and other miRNAs w/seed AUGUAAC) |
| 105.033 | 26.673 | 1.945 | -6.935 | 4.05E-12 | 2.95E-11 | miR-4669 (miRNAs w/seed GUGUCCG) |
| 128.646 | 33.701 | 1.9 | -7.557 | 4.13E-14 | 3.21E-13 | miR-344d-3p (and other miRNAs w/seed AUAUAAC) |
| 36.136 | 9.827 | 1.846 | -3.928 | 0.0000857 | 0.000419 | miR-513c-5p (and other miRNAs w/seed UCUCAAG) |
| 33.789 | 9.267 | 1.834 | -3.781 | 0.000156 | 0.00074 | miR-6503-3p (miRNAs w/seed GGACUAG) |
| 157.469 | 43.537 | 1.822 | -8.127 | 4.39E-16 | 3.62E-15 | miR-495-3p (and other miRNAs w/seed AACAAAC) |
| 246.853 | 69.567 | 1.795 | -10.07 | 7.53E-24 | 7.33E-23 | miR-6771-5p (miRNAs w/seed UCGGGAG) |
| 40.556 | 11.444 | 1.793 | -4.078 | 0.0000453 | 0.000229 | let-7f-2-3p (and other miRNAs w/seed UAUACAG) |
| 84.768 | 23.978 | 1.789 | -5.889 | 3.89E-09 | 2.52E-08 | miR-202-3p (and other miRNAs w/seed GAGGUAU) |
| 4554.879 | 1292.156 | 1.785 | -43.096 | 0 | 0 | miR-23a-3p (and other miRNAs w/seed UCACAUU) |
| 56.756 | 16.801 | 1.724 | -4.695 | 0.00000267 | 0.0000148 | miR-187-5p (miRNAs w/seed GCUACAA) |
| 49.944 | 14.819 | 1.72 | -4.398 | 0.0000109 | 0.0000573 | miR-1909-3p (and other miRNAs w/seed GCAGGGG) |
| 105.546 | 31.333 | 1.72 | -6.392 | 1.64E-10 | 1.11E-09 | miR-4783-5p (miRNAs w/seed GCGCGCC) |
| 338.376 | 104.91 | 1.657 | -11.148 | 7.35E-29 | 7.65E-28 | miR-1261 (miRNAs w/seed UGGAUAA) |
| 175.89 | 54.891 | 1.648 | -8.005 | 1.2E-15 | 9.76E-15 | miR-6716-5p (miRNAs w/seed GGGAAUG) |
| 65.219 | 20.56 | 1.633 | -4.843 | 0.00000128 | 0.00000721 | miR-3093-3p (and other miRNAs w/seed GUGGACA) |
| 411.65 | 130.12 | 1.629 | -12.147 | 5.94E-34 | 6.5E-33 | miR-3182 (miRNAs w/seed CUUCUGU) |
| 131.721 | 42.18 | 1.61 | -6.814 | 9.47E-12 | 6.77E-11 | miR-3614-5p (miRNAs w/seed CACUUGG) |
| 40.03 | 12.946 | 1.596 | -3.732 | 0.00019 | 0.00089 | miR-471-3p (and other miRNAs w/seed GAAAGGU) |
| 192.686 | 62.694 | 1.587 | -8.157 | 3.45E-16 | 2.9E-15 | miR-383-5p (miRNAs w/seed GAUCAGA) |
| 81.01 | 26.639 | 1.572 | -5.252 | 1.51E-07 | 0.000000907 | miR-873-3p (miRNAs w/seed GAGACUG) |
| 359.298 | 119.471 | 1.556 | -10.978 | 4.89E-28 | 5.07E-27 | miR-493-3p (miRNAs w/seed GAAGGUC) |
| 116.075 | 38.659 | 1.554 | -6.233 | 4.58E-10 | 3.09E-09 | miR-6763-3p (miRNAs w/seed UCCCCGG) |
| 51 | 17 | 1.552 | -4.129 | 0.0000364 | 0.000185 | miR-4719 (miRNAs w/seed CACAAAU) |
| 548.119 | 185.326 | 1.532 | -13.405 | 5.68E-41 | 6.92E-40 | miR-3651 (miRNAs w/seed AUAGCCC) |
| 212.017 | 72.731 | 1.511 | -8.253 | 1.54E-16 | 1.32E-15 | miR-543-3p (and other miRNAs w/seed AACAUUC) |
| 58.905 | 20.331 | 1.502 | -4.331 | 0.0000148 | 0.000077 | miR-1911-3p (miRNAs w/seed ACCAGGC) |
| 55.807 | 19.519 | 1.483 | -4.176 | 0.0000297 | 0.000152 | miR-6815-5p (and other miRNAs w/seed AGGUGGC) |
| 315.934 | 112.5 | 1.457 | -9.807 | 1.05E-22 | 1.01E-21 | miR-5189-3p (and other miRNAs w/seed GCCAACC) |
| 86.52 | 30.98 | 1.449 | -5.111 | 3.21E-07 | 0.00000189 | miR-453 (and other miRNAs w/seed GGUUGCC) |
| 46.815 | 16.807 | 1.445 | -3.752 | 0.000175 | 0.000828 | miR-8063 (miRNAs w/seed CAAAAUC) |
| 70.853 | 25.513 | 1.441 | -4.606 | 0.00000411 | 0.0000225 | miR-342-5p (and other miRNAs w/seed GGGGUGC) |
| 179.829 | 64.993 | 1.436 | -7.317 | 2.54E-13 | 1.9E-12 | miR-4716-3p (and other miRNAs w/seed AGGGGGA) |
| 226.23 | 82.509 | 1.423 | -8.15 | 3.62E-16 | 3.04E-15 | miR-4514 (and other miRNAs w/seed CAGGCAG) |
| 779.049 | 285.563 | 1.415 | -15.067 | 2.69E-51 | 3.57E-50 | miR-223-5p (miRNAs w/seed GUGUAUU) |
| 52.579 | 19.339 | 1.41 | -3.904 | 0.0000947 | 0.000461 | miR-4281 (miRNAs w/seed GGUCCCG) |
| 1175.49 | 433.25 | 1.407 | -18.429 | 7.69E-76 | 1.17E-74 | miR-139-5p (miRNAs w/seed CUACAGU) |
| 743.165 | 274.349 | 1.405 | -14.635 | 1.68E-48 | 2.17E-47 | miR-370-3p (and other miRNAs w/seed CCUGCUG) |
| 138.222 | 52.034 | 1.377 | -6.215 | 5.13E-10 | 3.44E-09 | miR-3195 (miRNAs w/seed GCGCCGG) |
| 54.393 | 20.947 | 1.344 | -3.828 | 0.000129 | 0.000622 | miR-3691-3p (miRNAs w/seed CCAAGUC) |
| 53.218 | 20.752 | 1.326 | -3.747 | 0.000179 | 0.000842 | miR-3103-5p (and other miRNAs w/seed GAGGGAG) |
| 165.779 | 64.782 | 1.323 | -6.602 | 4.07E-11 | 2.83E-10 | miR-664b-5p (miRNAs w/seed GGGCUAA) |
| 223.694 | 91.068 | 1.264 | -7.402 | 1.35E-13 | 1.02E-12 | miR-548an (miRNAs w/seed AAAGGCA) |
| 86.48 | 35.217 | 1.264 | -4.601 | 0.00000421 | 0.0000229 | miR-6918-5p (and other miRNAs w/seed GCUGAGG) |
| 673.776 | 275.242 | 1.259 | -12.806 | 1.51E-37 | 1.77E-36 | miR-1299 (miRNAs w/seed UCUGGAA) |
| 256.414 | 105.617 | 1.247 | -7.841 | 4.45E-15 | 3.58E-14 | miR-3473g (and other miRNAs w/seed AAAAGUG) |
| 581.875 | 240.593 | 1.242 | -11.771 | 5.48E-32 | 5.8E-31 | miR-432 (and other miRNAs w/seed CUUGGAG) |
| 96.89 | 40.914 | 1.211 | -4.711 | 0.00000247 | 0.0000137 | miR-1269a (and other miRNAs w/seed UGGACUG) |
| 1147.58 | 491.314 | 1.191 | -16.001 | 1.26E-57 | 1.75E-56 | miR-1270 (and other miRNAs w/seed UGGAGAU) |
| 496.804 | 213.813 | 1.184 | -10.475 | 1.12E-25 | 1.13E-24 | miR-3164 (and other miRNAs w/seed GUGACUU) |
| 1362.717 | 588.1 | 1.18 | -17.302 | 4.51E-67 | 6.47E-66 | miR-381-3p (and other miRNAs w/seed AUACAAG) |
| 191.378 | 83.75 | 1.16 | -6.396 | 1.6E-10 | 1.09E-09 | miR-374b-3p (miRNAs w/seed UUAGCAG) |
| 229.759 | 100.618 | 1.159 | -7.003 | 2.51E-12 | 1.83E-11 | miR-6073 (miRNAs w/seed GUAGUGA) |
| 65.2 | 28.767 | 1.148 | -3.703 | 0.000213 | 0.000998 | miR-1304-3p (and other miRNAs w/seed CUCACUG) |
| 94.814 | 41.994 | 1.142 | -4.448 | 0.00000867 | 0.0000458 | miR-139-3p (miRNAs w/seed GGAGACG) |
| 71195.5 | 31958.5 | 1.123 | -120.246 | 0 | 0 | miR-144-5p (miRNAs w/seed GAUAUCA) |
| 114.131 | 52.978 | 1.075 | -4.645 | 0.0000034 | 0.0000187 | miR-3150b-3p (and other miRNAs w/seed GAGGAGA) |
| 4085.957 | 1913.861 | 1.062 | -27.518 | 1.07E-166 | 2.34E-165 | miR-100-5p (and other miRNAs w/seed ACCCGUA) |
| 97.344 | 45.954 | 1.05 | -4.21 | 0.0000255 | 0.000132 | miR-6765-3p (miRNAs w/seed CACCUGG) |
| 643.651 | 304.177 | 1.049 | -10.814 | 2.96E-27 | 3.02E-26 | miR-379-5p (and other miRNAs w/seed GGUAGAC) |
| 4314.032 | 2049.185 | 1.041 | -27.835 | 1.65E-170 | 3.63E-169 | miR-28-3p (and other miRNAs w/seed ACUAGAU) |
| 811.01 | 387.524 | 1.033 | -11.987 | 4.15E-33 | 4.52E-32 | miR-1260a (and other miRNAs w/seed UCCCACC) |
| 150.691 | 72.612 | 1.021 | -5.117 | 0.00000031 | 0.00000184 | miR-150-3p (miRNAs w/seed UGGUACA) |
| 174.281 | 84.078 | 1.019 | -5.496 | 3.89E-08 | 0.000000241 | miR-4655-3p (miRNAs w/seed CCCUCGU) |

**72 Downregulated microRNA**

| **nonallergic Expression Intensity: FPKM** | **allergic Expression Intensity: FPKM** | **Log_2_FoldChange normalized** | **z-score** | **p-value** | **Expression False Discovery Rate (q-value)** | **Symbol** |
| --- | --- | --- | --- | --- | --- | --- |
| 10110.35 | 20043.79 | -1.02 | 59.645 | 0 | 0 | miR-362-5p (and other miRNAs w/seed AUCCUUG) |
| 99.392 | 199.418 | -1.037 | 6.032 | 1.62E-09 | 1.07E-08 | miR-34a-5p (and other miRNAs w/seed GGCAGUG) |
| 83.236 | 168.672 | -1.051 | 5.61 | 2.02E-08 | 0.000000126 | miR-186-3p (miRNAs w/seed CCCAAAG) |
| 115.1 | 234.1 | -1.057 | 6.636 | 3.22E-11 | 2.25E-10 | miR-548d-3p (and other miRNAs w/seed AAAAACC) |
| 141.678 | 288.919 | -1.061 | 7.394 | 1.42E-13 | 1.08E-12 | miR-151-5p (and other miRNAs w/seed CGAGGAG) |
| 503.75 | 1028.5 | -1.062 | 13.969 | 2.4E-44 | 3.04E-43 | miR-365-3p (and other miRNAs w/seed AAUGCCC) |
| 87977.48 | 180519.1 | -1.069 | 186.185 | 0 | 0 | miR-374b-5p (and other miRNAs w/seed UAUAAUA) |
| 4094.25 | 8411.314 | -1.071 | 40.224 | 0 | 0 | let-7i-3p (miRNAs w/seed UGCGCAA) |
| 884.208 | 1853.906 | -1.101 | 19.303 | 5.03E-83 | 7.99E-82 | miR-324-3p (miRNAs w/seed CCACUGC) |
| 1924.134 | 4044.545 | -1.104 | 28.589 | 9.33E-180 | 2.08E-178 | miR-18a-5p (and other miRNAs w/seed AAGGUGC) |
| 5731.011 | 12116.74 | -1.113 | 49.786 | 0 | 0 | miR-17-3p (and other miRNAs w/seed CUGCAGU) |
| 125 | 265.5 | -1.119 | 7.405 | 1.31E-13 | 1E-12 | miR-545-5p (miRNAs w/seed CAGUAAA) |
| 46.057 | 97.978 | -1.122 | 4.506 | 0.00000662 | 0.0000353 | miR-16-5p (and other miRNAs w/seed AGCAGCA) |
| 97.411 | 209.1 | -1.135 | 6.643 | 3.06E-11 | 2.15E-10 | miR-643 (miRNAs w/seed CUUGUAU) |
| 8766.85 | 18831.5 | -1.136 | 63.094 | 0 | 0 | miR-19b-3p (and other miRNAs w/seed GUGCAAA) |
| 659.906 | 1420.293 | -1.138 | 17.361 | 1.62E-67 | 2.35E-66 | miR-501-5p (miRNAs w/seed AUCCUUU) |
| 138.208 | 299.795 | -1.15 | 8.04 | 9.01E-16 | 7.37E-15 | miR-6513-5p (miRNAs w/seed UUGGGAU) |
| 81 | 177.066 | -1.161 | 6.227 | 4.77E-10 | 3.21E-09 | miR-182-3p (miRNAs w/seed GGUUCUA) |
| 104.747 | 230.149 | -1.168 | 7.135 | 9.7E-13 | 7.14E-12 | miR-4690-5p (miRNAs w/seed AGCAGGC) |
| 394 | 867.062 | -1.17 | 13.87 | 9.65E-44 | 1.2E-42 | miR-329-3p (and other miRNAs w/seed ACACACC) |
| 2659.365 | 6001.546 | -1.207 | 37.387 | 6E-306 | 1E-304 | miR-505-3p (miRNAs w/seed GUCAACA) |
| 141.584 | 321.233 | -1.214 | 8.693 | 3.53E-18 | 3.13E-17 | miR-188-5p (and other miRNAs w/seed AUCCCUU) |
| 60.162 | 137 | -1.22 | 5.697 | 1.22E-08 | 7.71E-08 | miR-4768-5p (and other miRNAs w/seed UUCUCUC) |
| 291.728 | 675.811 | -1.245 | 12.854 | 8.2E-38 | 9.62E-37 | miR-548aa (and other miRNAs w/seed AAAACCA) |
| 147.083 | 348 | -1.275 | 9.4 | 5.47E-21 | 5.1E-20 | miR-15a-3p (miRNAs w/seed AGGCCAU) |
| 147.656 | 354.561 | -1.296 | 9.611 | 7.17E-22 | 6.78E-21 | miR-3136-3p (and other miRNAs w/seed GGCCCAA) |
| 44.799 | 107.688 | -1.298 | 5.302 | 1.15E-07 | 0.000000697 | miR-4689 (and other miRNAs w/seed UGAGGAG) |
| 547 | 1318 | -1.301 | 18.585 | 4.23E-77 | 6.51E-76 | miR-21-5p (and other miRNAs w/seed AGCUUAU) |
| 48.077 | 121.011 | -1.364 | 5.84 | 5.22E-09 | 3.36E-08 | miR-3194-3p (miRNAs w/seed GCUCUGC) |
| 36680.66 | 93548.11 | -1.383 | 164.13 | 0 | 0 | miR-30c-5p (and other miRNAs w/seed GUAAACA) |
| 194.484 | 502.612 | -1.402 | 12.154 | 5.49E-34 | 6.05E-33 | miR-3660 (and other miRNAs w/seed CUGACAG) |
| 260 | 698.7 | -1.459 | 14.761 | 2.63E-49 | 3.42E-48 | miR-1277-5p (miRNAs w/seed AAUAUAU) |
| 2963.826 | 8078.925 | -1.479 | 50.719 | 0 | 0 | miR-500a-5p (miRNAs w/seed AAUCCUU) |
| 18.038 | 50.043 | -1.505 | 4.042 | 0.0000529 | 0.000263 | miR-5003-5p (miRNAs w/seed CACAACA) |
| 197.591 | 557.424 | -1.529 | 13.651 | 1.99E-42 | 2.45E-41 | miR-6511b-5p (and other miRNAs w/seed UGCAGGC) |
| 191.653 | 548.258 | -1.549 | 13.668 | 1.57E-42 | 1.93E-41 | miR-6511a-5p (and other miRNAs w/seed AGGCAGA) |
| 164.634 | 477.917 | -1.57 | 12.888 | 5.23E-38 | 6.22E-37 | miR-320b (and other miRNAs w/seed AAAGCUG) |
| 1119.346 | 3297.452 | -1.591 | 34.186 | 3.94E-256 | 9.78E-255 | miR-1307-5p (miRNAs w/seed CGACCGG) |
| 551.833 | 1652.188 | -1.615 | 24.454 | 4.51E-132 | 9E-131 | miR-3617-5p (and other miRNAs w/seed AAGACAU) |
| 3775.111 | 11538.83 | -1.644 | 65.483 | 0 | 0 | miR-550a-3p (and other miRNAs w/seed GUCUUAC) |
| 3218.31 | 9859.263 | -1.648 | 60.615 | 0 | 0 | miR-130a-3p (and other miRNAs w/seed AGUGCAA) |
| 31 | 97.083 | -1.679 | 6.097 | 1.08E-09 | 7.16E-09 | miR-153-3p (miRNAs w/seed UGCAUAG) |
| 130.949 | 411.551 | -1.685 | 12.581 | 2.7E-36 | 3.08E-35 | miR-3667-5p (miRNAs w/seed AAGACCC) |
| 361.326 | 1173.459 | -1.732 | 21.662 | 4.7E-104 | 8.43E-103 | miR-12197-3p (and other miRNAs w/seed CGGGGCU) |
| 149.942 | 486.959 | -1.732 | 13.954 | 2.96E-44 | 3.7E-43 | miR-762 (and other miRNAs w/seed GGGCUGG) |
| 63.643 | 210.577 | -1.759 | 9.275 | 1.77E-20 | 1.63E-19 | miR-1537-3p (miRNAs w/seed AAACCGU) |
| 42.321 | 141.408 | -1.773 | 7.643 | 2.12E-14 | 1.67E-13 | miR-937-5p (miRNAs w/seed UGAGUCA) |
| 42.127 | 145.155 | -1.817 | 7.877 | 3.36E-15 | 2.71E-14 | miR-1273h-5p (and other miRNAs w/seed UGGGAGG) |
| 20.472 | 72.431 | -1.855 | 5.644 | 1.67E-08 | 0.000000105 | miR-1587 (and other miRNAs w/seed UGGGCUG) |
| 95.3 | 352 | -1.918 | 12.72 | 4.59E-37 | 5.29E-36 | miR-545-3p (miRNAs w/seed CAGCAAA) |
| 8.724 | 32.644 | -1.936 | 3.899 | 0.0000967 | 0.00047 | miR-149-3p (and other miRNAs w/seed GGGAGGG) |
| 40.62 | 152.461 | -1.941 | 8.438 | 3.22E-17 | 2.78E-16 | miR-3130-5p (and other miRNAs w/seed ACCCAGU) |
| 95.671 | 359.527 | -1.942 | 12.966 | 1.91E-38 | 2.28E-37 | miR-6768-5p (miRNAs w/seed ACACAGG) |
| 327.297 | 1257.271 | -1.974 | 24.507 | 1.23E-132 | 2.48E-131 | miR-6089 (miRNAs w/seed GAGGCCG) |
| 260.512 | 1008.562 | -1.985 | 22.032 | 1.42E-107 | 2.64E-106 | miR-103-1-5p (and other miRNAs w/seed GCUUCUU) |
| 76.667 | 302.19 | -2.011 | 12.163 | 4.92E-34 | 5.44E-33 | miR-196a-5p (and other miRNAs w/seed AGGUAGU) |
| 70.801 | 282.69 | -2.03 | 11.834 | 2.6E-32 | 2.8E-31 | miR-6132 (and other miRNAs w/seed GCAGGGC) |
| 8.674 | 34.993 | -2.045 | 4.183 | 0.0000287 | 0.000148 | miR-1237-5p (and other miRNAs w/seed GGGGGCG) |
| 17.258 | 71.594 | -2.085 | 6.059 | 1.37E-09 | 9.01E-09 | miR-10396b-5p (and other miRNAs w/seed GGCGGGG) |
| 8.438 | 36.256 | -2.136 | 4.378 | 0.000012 | 0.0000625 | miR-1266-5p (and other miRNAs w/seed CUCAGGG) |
| 24.006 | 105.238 | -2.165 | 7.522 | 5.39E-14 | 4.16E-13 | miR-143-3p (and other miRNAs w/seed GAGAUGA) |
| 12.408 | 54.743 | -2.174 | 5.44 | 5.34E-08 | 0.000000327 | miR-4292 (and other miRNAs w/seed CCCUGGG) |
| 142.421 | 639.449 | -2.199 | 18.726 | 3.07E-78 | 4.75E-77 | miR-33-5p (and other miRNAs w/seed UGCAUUG) |
| 5.45 | 25.13 | -2.238 | 3.752 | 0.000176 | 0.000828 | miR-3473h-5p (and other miRNAs w/seed AGGGGCU) |
| 7.773 | 36.408 | -2.26 | 4.544 | 0.00000552 | 0.0000297 | miR-497-3p (and other miRNAs w/seed CAAACCA) |
| 410.294 | 2245.511 | -2.485 | 37.734 | 0 | 0 | miR-122-5p (miRNAs w/seed GGAGUGU) |
| 10.6 | 62 | -2.581 | 6.405 | 1.51E-10 | 1.03E-09 | miR-133a-3p (and other miRNAs w/seed UUGGUCC) |
| 135.29 | 881.981 | -2.737 | 24.935 | 3.08E-137 | 6.41E-136 | miR-1234-3p (and other miRNAs w/seed CGGCCUG) |
| 41.773 | 275.059 | -2.752 | 13.964 | 2.6E-44 | 3.27E-43 | miR-3083-5p (and other miRNAs w/seed GGCUGGG) |
| 3.667 | 25.688 | -2.841 | 4.338 | 0.0000144 | 0.0000748 | miR-1288-5p (miRNAs w/seed CAGAUCA) |
| 15.394 | 179.089 | -3.573 | 12.689 | 6.79E-37 | 7.78E-36 | miR-1-3p (and other miRNAs w/seed GGAAUGU) |
| 0 | 21.929 | -4.71 | 4.807 | 0.00000154 | 0.00000865 | miR-1245b-5p (and other miRNAs w/seed AGGCCUU) |
