# Supplementary material for: Differentially expressed microRNAs in peripheral blood cell are associated with downregulated expression of IgE in nonallergic childhood asthma
Source: Sci Rep. 2023 Apr 19;13:6381. doi: 10.1038/s41598-023-33663-5 (PMC10115804; doi:10.1038/s41598-023-33663-5)
Supplement: Supplementary file 9 — Supplementary Information 9. [file 41598_2023_33663_MOESM9_ESM.docx]

**Supplement Table 3:** mRNA-mRNA interaction between predicted mRNA target of differentially expressed microRNAs.

| **From Molecule(s)** | **Relationship Type** | **To Molecule(s)** |
| --- | --- | --- |
| ADRB2 | expression | IL10 |
| ADRB2 | regulation of binding | ADRB2 |
| ALOX15 | expression | ALOX15 |
| ALOX15 | expression | NOS2 |
| BDNF | regulation of binding | NTRK2 |
| CCL5 | expression | CXCL8 |
| CCL5 | expression | IL1B |
| CCL5 | expression | MMP9 |
| CCL5 | localization | IL4 |
| CD14 | expression | CCL5 |
| CD14 | expression | IL10 |
| CD14 | expression | IL1B |
| CD14 | localization | CXCL8 |
| CD14 | localization | IL10 |
| CXCL8 | expression | CXCL8 |
| CXCL8 | expression | IL1B |
| CXCL8 | expression | MMP9 |
| CXCL8 | localization | MMP9 |
| CYSLTR2 | expression | CYSLTR1 |
| ERBB2 | expression | CXCL8 |
| ERBB2 | expression | IL1A |
| ERBB2 | expression | IL2 |
| ERBB2 | expression | NRG1 |
| ERBB2 | regulation of binding | ERBB2 |
| FCER2 | activation | NOS2 |
| FCER2 | expression | IgE |
| FCER2 | expression | NOS2 |
| FCER2 | transcription | CXCL8 |
| HAVCR2 | expression | IL2 |
| IL10 | expression | CCL5 |
| IL10 | expression | CD14 |
| IL10 | expression | CXCL8 |
| IL10 | expression | IL10 |
| IL10 | expression | IL1B |
| IL10 | expression | IL4 |
| IL10 | expression | IgE |
| IL10 | expression | NOS2 |
| IL10 | expression | TLR4 |
| IL10 | localization | IL1B |
| IL10 | transcription | IL10 |
| IL13 | activation | STAT6 |
| IL13 | expression | ALOX15 |
| IL13 | expression | BDNF |
| IL13 | expression | C3AR1 |
| IL13 | expression | CCL11 |
| IL13 | expression | CD14 |
| IL13 | expression | CXCL8 |
| IL13 | expression | CYSLTR1 |
| IL13 | expression | FCER2 |
| IL13 | expression | IL10 |
| IL13 | expression | IL1B |
| IL13 | expression | TSLP |
| IL13 | molecular cleavage | IL1B |
| IL13 | phosphorylation | STAT6 |
| IL13 | regulation of binding | CCL11 |
| IL13 | regulation of binding | STAT6 |
| IL13 | transcription | CCL11 |
| IL13 | translocation | STAT6 |
| IL1A | expression | CCL5 |
| IL1A | expression | CXCL8 |
| IL1A | expression | IL1A |
| IL1A | expression | IL1B |
| IL1A | expression | NOS2 |
| IL1A | expression | TGFB1 |
| IL1A | localization | CXCL8 |
| IL1A | localization | MMP9 |
| IL1A | regulation of binding | CXCL8 |
| IL1B | expression | CCL11 |
| IL1B | expression | CCL5 |
| IL1B | expression | CXCL8 |
| IL1B | expression | IL10 |
| IL1B | expression | IL1A |
| IL1B | expression | IL1B |
| IL1B | expression | MMP9 |
| IL1B | expression | NOS2 |
| IL1B | expression | TGFB1 |
| IL1B | expression | TLR4 |
| IL1B | localization | CCL11 |
| IL1B | localization | CCL5 |
| IL1B | localization | CXCL8 |
| IL1B | regulation of binding | CXCL8 |
| IL1B | transcription | CCL11 |
| IL1B | transcription | CCL5 |
| IL1RAP | expression | CD14 |
| IL1RAP | expression | CXCL8 |
| IL2 | expression | IL10 |
| IL2 | expression | IL13 |
| IL2 | expression | IL1B |
| IL2 | expression | IL7R |
| IL2 | localization | IL10 |
| IL2 | localization | IL2 |
| IL2 | localization | IL4 |
| IL3 | expression | CD14 |
| IL3 | expression | IL13 |
| IL3 | expression | IL1B |
| IL3 | expression | IL4 |
| IL3 | expression | MS4A2 |
| IL33 | expression | CXCL8 |
| IL33 | expression | IL13 |
| IL33 | expression | IL1B |
| IL33 | expression | IL1RL1 |
| IL33 | expression | IL2 |
| IL33 | expression | IL4 |
| IL33 | localization | IL4 |
| IL4 | activation | ALOX15 |
| IL4 | expression | ALOX15 |
| IL4 | expression | CCL11 |
| IL4 | expression | CCL17 |
| IL4 | expression | CD14 |
| IL4 | expression | CXCL8 |
| IL4 | expression | CYSLTR1 |
| IL4 | expression | CYSLTR2 |
| IL4 | expression | IL10 |
| IL4 | expression | IL1B |
| IL4 | expression | IL2 |
| IL4 | expression | IL4 |
| IL4 | expression | IL4R |
| IL4 | expression | IgE |
| IL4 | expression | MMP9 |
| IL4 | expression | TNFSF4 |
| IL4 | expression | TSLP |
| IL4 | localization | CCL17 |
| IL4 | localization | FCER2 |
| IL4 | localization | IgE |
| IL4 | phosphorylation | STAT6 |
| IL4 | regulation of binding | STAT6 |
| IL4 | translocation | STAT6 |
| IL4R | phosphorylation | STAT6 |
| IL7R | expression | CYSLTR2 |
| ITGB3 | expression | ITGB3 |
| Ige | expression | CCL5 |
| Ige | expression | IL13 |
| Ige | expression | IL4 |
| Ige | localization | IL4 |
| MMP9 | expression | TGFB1 |
| NGF | localization | IL1B |
| NOS2 | expression | NOS2 |
| NRG1 | expression | NRG1 |
| NRG1 | phosphorylation | ERBB2 |
| PDE4D | localization | IL2 |
| STAT6 | localization | CCL17 |
| TGFB1 | expression | CCL11 |
| TGFB1 | expression | CCL5 |
| TGFB1 | expression | CXCL8 |
| TGFB1 | expression | IL1B |
| TGFB1 | expression | IL1RL1 |
| TGFB1 | expression | ITGB3 |
| TGFB1 | expression | MMP9 |
| TGFB1 | expression | TGFB1 |
| TGFB1 | expression | TLR4 |
| TGFB1 | localization | MMP9 |
| TGFB1 | localization | TGFB1 |
| TGFB1 | phosphorylation | STAT6 |
| TGFB1 | regulation of binding | CCL11 |
| TGFB1 | regulation of binding | STAT6 |
| TGFB1 | transcription | CCL11 |
| TGFB1 | translocation | STAT6 |
| TLR4 | expression | CCL5 |
| TLR4 | expression | CXCL8 |
| TLR4 | expression | IL10 |
| TLR4 | expression | IL13 |
| TLR4 | expression | IL1B |
| TLR4 | expression | IL4 |
| TLR4 | expression | NOS2 |
| TLR4 | expression | TSLP |
| TLR4 | localization | CCL5 |
| TLR4 | localization | CD14 |
| TLR4 | localization | CXCL8 |
| TLR4 | localization | IL10 |
| TLR4 | localization | IL1B |
| TSLP | expression | CCL17 |
| TSLP | expression | IL10 |
| TSLP | expression | TNFSF4 |
| TSLP | localization | CCL17 |
| TSLP | transcription | CCL11 |
| TSLP | transcription | CXCL8 |
